# TGF-β-Induced Transcription Sustains Amoeboid Melanoma Migration and Dissemination

**DOI:** 10.1016/j.cub.2015.09.054

**Published:** 2015-11-16

**Authors:** Gaia Cantelli, Jose L. Orgaz, Irene Rodriguez-Hernandez, Panagiotis Karagiannis, Oscar Maiques, Xavier Matias-Guiu, Frank O. Nestle, Rosa M. Marti, Sophia N. Karagiannis, Victoria Sanz-Moreno

**Affiliations:** 1 Tumour Plasticity Laboratory, Randall Division of Cell and Molecular Biophysics, New Hunt’s House, Guy’s Campus, King’s College London, London SE11UL, UK; 2 St. John’s Institute of Dermatology, Division of Genetics and Molecular Medicine, Faculty of Life Sciences and Medicine, King’s College London and National Institute for Health Research (NIHR) Biomedical Research Centre at Guy’s and St Thomas’ Hospitals, King’s College London, Guy’s Campus, London SE1 9RT, UK; 3 Department of Oncology, Haematology and Stem Cell Transplantation, University Hospital of Hamburg Eppendorf, Hamburg 20246, Germany; 4 Department of Pathology and Molecular Genetics, Hospital Universitari Arnau de Vilanova, University of Lleida, IRBLleida, Lleida 25198, Spain; 5 Department of Dermatology, Hospital Universitari Arnau de Vilanova, University of Lleida, IRBLleida, Lleida 25198, Spain

## Abstract

Cell migration underlies metastatic dissemination of cancer cells, and fast “amoeboid” migration in the invasive fronts of tumors is controlled by high levels of actomyosin contractility. How amoeboid migration is regulated by extracellular signals and sustained over time by transcriptional changes is not fully understood. Transforming growth factor β (TGF-β) is well known to promote epithelial-to-mesenchymal transition (EMT) and contribute to metastasis, but melanocytes are neural crest derivatives that have undergone EMT during embryonic development. Surprisingly, we find that in melanoma, TGF-β promotes amoeboid features such as cell rounding, membrane blebbing, high levels of contractility, and increased invasion. Using genome-wide transcriptomics, we find that amoeboid melanoma cells are enriched in a TGF-β-driven signature. We observe that downstream of TGF-β, SMAD2 and its adaptor CITED1 control amoeboid behavior by regulating the expression of key genes that activate contractile forces. Moreover, CITED1 is highly upregulated during melanoma progression, and its high expression is associated with poor prognosis. CITED1 is coupled to a contractile-rounded, amoeboid phenotype in a panel of 16 melanoma cell lines, in mouse melanoma xenografts, and in 47 human melanoma patients. Its expression is also enriched in the invasive fronts of lesions. Functionally, we show how the TGF-β-SMAD2-CITED1 axis promotes different steps associated with progression: melanoma detachment from keratinocytes, 2D and 3D migration, attachment to endothelial cells, and in vivo lung metastatic initial colonization and outgrowth. We propose a novel mechanism by which TGF-β-induced transcription sustains actomyosin force in melanoma cells and thereby promotes melanoma progression independently of EMT.

## Highlights

• TGF-β-SMAD promotes amoeboid migration in melanoma
• Downstream of TGF-β, the adaptor CITED1 controls actomyosin contractility
• Amoeboid features correlate with CITED1 levels in cell lines, xenografts, and patients
• TGF-β-SMAD-CITED1 transcriptional network controls melanoma metastatic ability

## Graphical Abstract

**Figure:**
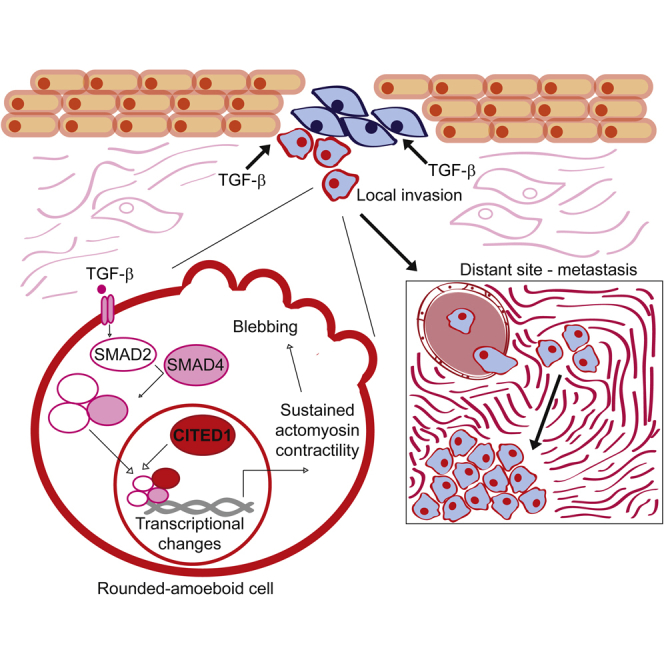

## Teaser

Cantelli et al. find that, in melanoma, TGF-β-SMAD-CITED1 controls amoeboid behavior through activation of a transcriptional program. As a result, melanoma cells detach from keratinocytes, increase their invasive potential, and efficiently colonize the lung. This is a new function of TGF-β, independent of epithelial-to-mesenchymal transition.

## Introduction

The transforming growth factor β (TGF-β) signaling pathway plays a major role in the regulation of the epithelial-to-mesenchymal transition (EMT), which governs morphogenesis and the progression of carcinomas [1]. TGF-β signaling acts as a tumor promoter in advanced epithelial tumors and drives metastasis [2] by favoring EMT, proliferation, dissemination, angiogenesis, and tumor escape from immune surveillance [3, 4, 5]. TGF-β ligands bind to the type II TGF-β receptor, in turn, activating the type I receptor. The type I receptor phosphorylates downstream effectors SMAD2 and SMAD3, which then associate with SMAD4 [6, 7]. The SMAD2/3-SMAD4 complex accumulates in the cell nucleus, where it regulates the transcription of various target genes.

SMAD-mediated transcription is fine-tuned by a variety of co-factors, co-activators (or co-repressors), and adaptors [8]. CITED1 (also known as MSG1) [9] is a well-known adaptor protein for this complex, and, as such, it acts as a specificity factor directing the activity of TGF-β-driven transcription. It does so by binding to SMAD4 and to the non-specific co-activator p300 and promoting their interaction [10]. CITED1 has been linked to melanocyte pigmentation [9], and it has been shown to play a role in development [11] and in mediating stemness [12]. CITED1 deregulation is associated with a variety of cancers [9, 11, 12, 13, 14, 15]. However, its connection to invasive behavior remains unknown to date.

Melanoma is the most serious type of skin cancer due to its high metastatic ability [16]. Skin melanocytes are found in the basal layer of the epidermis and derive from highly motile neural crest progenitors [17], which colonize the body during development. Neural crest cells undergo EMT early in development, migrate throughout the embryo, and subsequently differentiate into a variety of cell types, including melanocytes. The invasive and metastatic potential of melanoma cells thus reflects their ability to revert to a less differentiated, neural crest-like phenotype [18].

Melanoma cells display an inherent ability to switch between modes of migration [19, 20]. Among different migratory strategies, rounded-amoeboid behavior is characterized by rounded morphology as well as blebs as functional protrusions [21], low levels of adhesion [22, 23], and high levels of actomyosin contractility, driven by Rho-ROCK [24] and JAK-STAT3 signaling [25, 26]. Moreover, some types of amoeboid migration have been reported to be independent of transcriptional regulation [22, 23]. Rounded-amoeboid behavior is prominent in the invasive fronts of melanomas and breast cancer tumors in animal models [19, 26, 27] and in human melanoma lesions [25, 26]. The interface between the tumor invasive front and the stroma favors TGF-β signaling in a paracrine and autocrine manner [28]. In melanoma, TGF-β-induced genes have been detected in the invasive fronts of lesions [29].

In the current study, we have explored the role of TGF-β-dependent transcription in regulating melanoma migratory strategies using both in vitro and in vivo approaches. We find that TGF-β, SMAD2, and its adaptor protein CITED1 control amoeboid migration, independently from the role of TGF-β in promoting EMT.

## Results

### TGF-β Promotes Amoeboid Features

**Figure 1:**
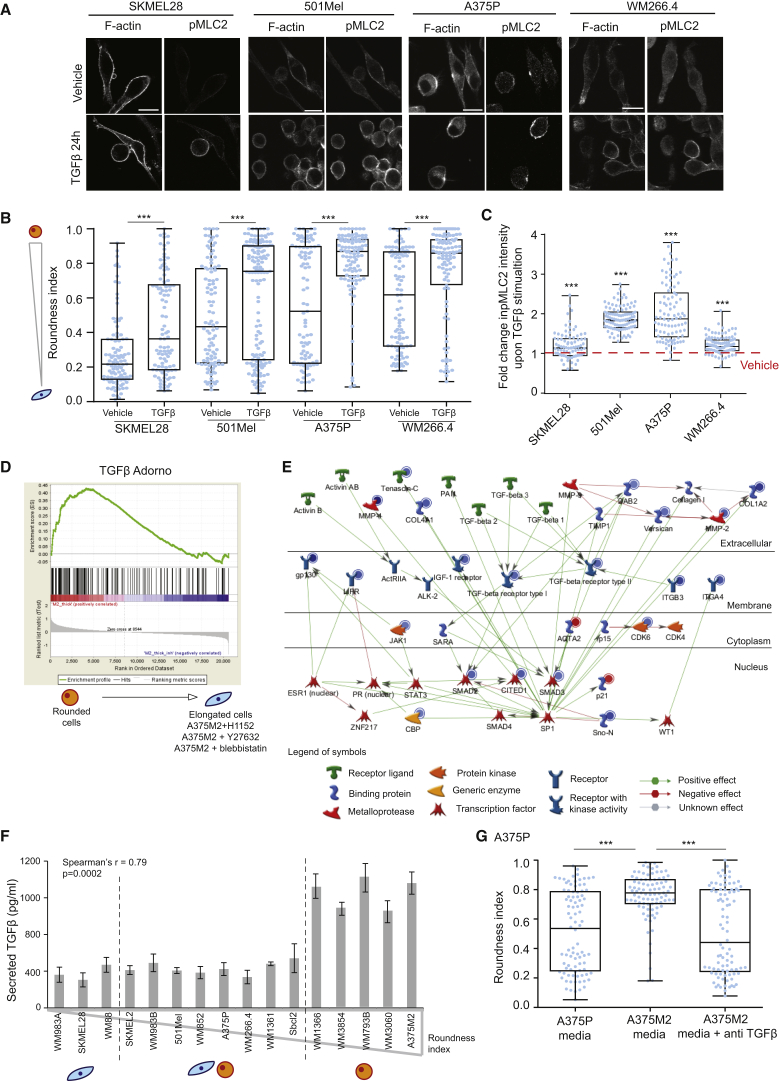
TGF-β Promotes Amoeboid Features (A) Representative confocal images of phospho-MLC2 (pMLC2) immunostaining of SKMEL28, 501Mel, A375P, and WM266.4 cells upon 24 hr stimulation with TGF-β in serum-free media. F-actin staining is also shown. Scale bar, 20 μm. (B) Cell morphology (roundness index) of SKMEL28, 501Mel, A375P, and WM266.4 cells upon 24 hr stimulation with TGF-β. Dots represent single cells from three independent experiments (n = 3 experiments; n = 100 cells). (C) Quantification of pMLC2 levels from immunostaining in confocal images from (A). Dots represent single cells from three independent experiments (n = 3; n = 100). Indicated statistics are versus vehicle. (D) Gene set enrichment analysis (GSEA) plot of gene expression microarray analysis comparing untreated A375M2 cells and contractility-inhibited A375M2 cells (H1152, Y27632, blebbistatin). GSEA plot shows the enrichment score on the y axis for genes that are known to be regulated by TGF-β stimulation [30]. Genes are ordered on the x axis according to their GSEA enrichment score. Enrichment plots show upregulation of TGF-β-regulated genes in rounded, more contractile cells compared to less contractile cells. Nominal p value <0.001. (E) Significantly enriched network following network enrichment analysis using MetaCore. Following contractility inhibition, downregulated genes are marked with blue circles, and upregulated genes are marked with red circles. Legend indicates the meaning of the different shapes representing different molecular categories. (F) Levels of secreted TGF-β by ELISA in a panel of 16 melanoma cell lines of varying morphology (n = 3). Cell lines are arranged by morphology; correlation between average roundness index and secreted TGF-β level is also shown. (G) Cell morphology (roundness index) of A375P cells treated with conditioned media from A375P cells of A375M2 cells with and without the addition of a TGF-β blocking antibody (n = 3; n = 60). Dots represent single cells from three independent experiments. n.s., not significant, ^∗^p < 0.05, ^∗∗^p < 0.01, ^∗∗∗^p < 0.001. Graphs show mean ± SEM. Unpaired t test (B and C); Tukey’s post-test following one-way ANOVA (G); Spearman’s correlation (F). See also Figure S1.

To investigate a possible role of TGF-β in controlling cytoskeletal features in melanoma, we treated different melanoma cell lines with TGF-β. Interestingly, all melanoma cell lines tested (SKMEL28, 501MEL, A375P, and WM266.4) increased their roundness index and their levels of actomyosin contractility (measured as MLC2 phosphorylation) after treatment with TGF-β (Figures 1A–1C; Figure S1A).

Since TGF-β is a transcriptional regulator, we analyzed the transcriptional signature of rounded and highly contractile A375M2 melanoma cells compared to A375M2 cells treated with contractility inhibitors (ROCK inhibitors H1152, Y27632, and blebbistatin, an inhibitor of Myosin II ATPase) or less contractile A375P cells [26]. Using gene set enrichment analysis (GSEA), we found a significant enrichment of TGF-β-regulated genes in rounded and highly contractile cells (Figure 1D). Furthermore, performing Network Enrichment Analysis with GeneGo Metacore, we found that in rounded contractile cells there was a significant enrichment in a network of genes centered on SMAD signaling (Figures 1E and S1B). Enriched genes included TGF-βRI, TGF-βRII, SMAD2, SMAD3, and CITED1.

Since TGF-β itself was not regulated at the mRNA level, we measured secreted TGF-β by ELISA in a panel of 16 melanoma cell lines of varying degrees of rounding (Figures 1F and S1C). Cell morphology in the panel was assessed in cells plated on a thick layer of collagen I [25, 26]. We found a striking correlation between roundness and secreted levels of TGF-β (Figure 1F). As secreted factors work in a concentration-dependent manner, only cell populations with a roundness index higher than 0.75 secreted enough TGF-β levels to sustain rounding (Figure S1C). These data could suggest that TGF-β controls amoeboid features in an autocrine manner.

In order to understand whether TGF-β can play a role in controlling cell morphology in a paracrine manner, we treated low-TGF-β, low-contractility cells (A375P) with conditioned media from high-TGF-β, high-contractility cells (A375M2). While A375M2-derived media induced rounding in A375P cells, this effect was lost in the presence of a TGF-β-neutralizing antibody (Figures 1G and S1D). These results suggest a possible paracrine action of TGF-β by regulating amoeboid features in melanoma.

### Downstream of TGF-β, SMAD2 Controls Cytoskeletal Actomyosin in Melanoma

**Figure 2:**
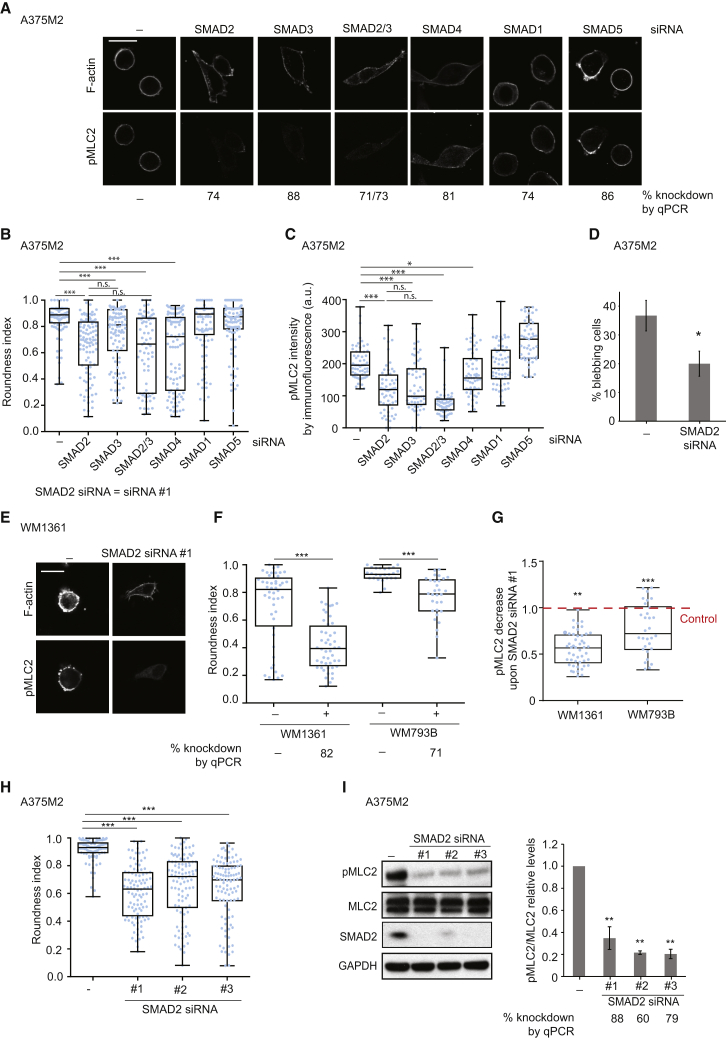
Downstream of TGF-β, SMAD2 Controls Cytoskeletal Actomyosin in Melanoma (A) Representative confocal images of phospho-MLC2 (pMLC2) immunostaining of A375M2 upon SMAD2, SMAD3, SMAD2/3, SMAD4, SMAD1, and SMAD5 knockdown. F-actin staining is also shown. Scale bar, 20 μm. Percentage knockdown by quantitative RT-PCR (qPCR) indicated below the images. (B) Cell morphology (roundness index) of A375M2 upon SMAD2, SMAD3, SMAD2/3, SMAD4, SMAD1, and SMAD5 knockdown. Dots represent individual cells from three independent experiments (n = 3 experiments; n = 100 cells). (C) Quantification of pMLC2 levels from immunostaining in confocal images from (A). Dots represent individual cells from 3 independent experiments (n = 3; n = 60). (D) Percentage of blebbing cells in A375M2 cells on collagen I upon SMAD2 siRNA (#1) knockdown (n = 3). (E) Representative confocal images of pMLC2 immunostaining of WM1361 cells upon SMAD2 siRNA (#1) knockdown. F-actin staining is also shown. Scale bar, 20 μm. (F) Cell morphology (roundness index) of WM1361 and WM793B cells upon SMAD2 siRNA (#1) knockdown. Dots represent individual cells from three independent experiments (n = 3; n = 30). Percentage knockdown by qPCR is indicated below. (G) Quantification of pMLC2 levels from immunostaining in confocal images from (F). Dots represent individual cells from three independent experiments (n = 3; n = 30). Indicated statistics are versus control. (H) Cell morphology (roundness index) of A375M2 cells upon SMAD2 siRNA knockdown with SmartPool (#1) and two individual OnTargetplus oligos (#2 and #3). Dots represent individual cells from three independent experiments (n = 3; n = 100). (I) Representative immunoblots (left) and quantification (right) of pMLC2, MLC2, and SMAD2 of A375M2 cells on collagen I upon SMAD2 siRNA #1 knockdown (n = 3). Percentage of knockdown by qPCR is shown below. n.s., not significant, ^∗^p < 0.05, ^∗∗^p < 0.01, ^∗∗∗^p < 0.001. Graphs show mean ± SEM. Tukey’s post-test following one-way ANOVA (B, C, H, and I); unpaired t test (D, F, and G).

Downstream of TGF-β, SMADs drive transcriptional programs important for EMT in carcinomas. SMAD2/3 and SMAD1/5 are known as R-SMADs and are activated respectively by TGF-β/Activin/Nodal or BMP signals [31, 32], while SMAD4 acts with both sets of SMADs [6]. We determined which members of the SMAD family control the actomyosin cytoskeleton in melanoma by knocking down its members individually via RNAi. Out of all the SMADs tested (SMAD1, 2, 3, 4, and 5), we observed significant loss of roundness (Figures 2A and 2B) and decreased phospho-MLC2 levels (Figures 2A and 2C) after depletion of SMAD2, SMAD3, and, to a lesser extent, SMAD4. SMAD2 and SMAD3 simultaneous depletion did not result in a cumulative effect and resulted in loss of rounding and reduced contractility similar to those observed after SMAD2 knockdown alone (Figures 2A–2C).

We observed the strongest effects after depleting SMAD2, which resulted in loss of cell rounding, decreased MLC2 activity, and loss of blebbing (Figures 2A–2D). We verified these results using two other melanoma cell lines, WM1361 and WM793B (Figures 2E–2G), and using several oligonucleotides against SMAD2 (Figures 2H and 2I). These results show that SMAD2 is required to sustain rounding and actomyosin cytoskeletal activity in melanoma cells.

### Downstream of TGF-β, CITED1 Is Associated with Contractile Features

**Figure 3:**
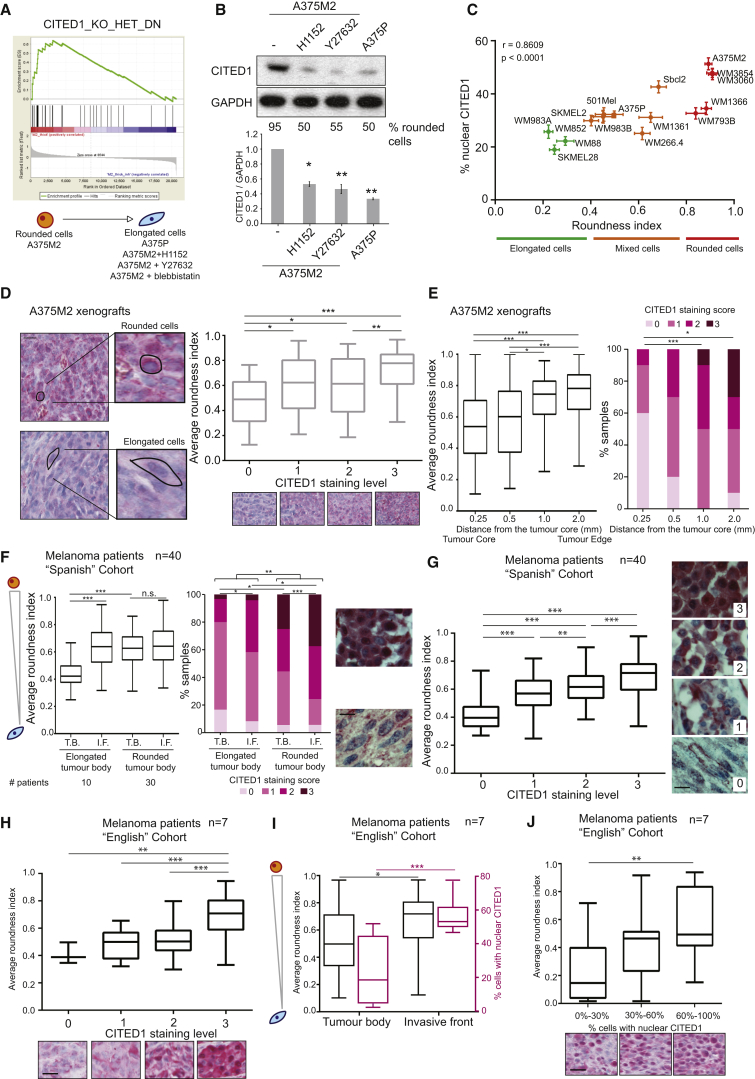
Downstream of TGF-β CITED1 Is Associated with Contractile Features (A) Gene set enrichment analysis (GSEA) plot of gene expression microarray analysis comparing untreated A375M2 cells and contractility-inhibited A375M2 cells (H1152, Y27632, blebbistatin) or A375P cells. Enrichment plots show upregulation of CITED1-regulated genes in rounded, more contractile cells compared to less contractile cells. Nominal p value <0.001. (B) Representative immunoblot (top) and quantification (bottom) of CITED1 in A375M2 upon ROCK inhibition and A375P cells on collagen I. For each condition, percentage of rounded cells on top of collagen I is shown below the blot. (C) Scatterplot of the roundness index and percentage of nuclear CITED1 staining correlation analysis in a panel of 16 melanoma cell lines. Dots represent mean expression, and horizontal and vertical error bars are ±SEM of x and y variables, respectively. Correlation between roundness index and percentage of nuclear CITED1 (Spearman’s r) is also shown. Data also represented in Figure S2B. (D) (left) Representative images of different levels of CITED1 immunostaining in mouse xenografts of A375M2 cells. Images highlight a typically rounded cell and a typically elongated cell. Scale bar, 20 μm. (right) Quantification of average roundness index of A375M2 xenografts classified by level of CITED1 staining (n = 5 xenografts; n = 700 cells). (E) (left) Boxplot showing the average roundness cell index in fields taken at increasing distance from the center of the xenograft. (right) Distribution of CITED1 levels in fields taken at increasing distance from the center of the xenograft. (F) (left) Average roundness index and (right) distribution of CITED1 staining for melanoma cells in the tumor body (T.B.) or the invasive front (I.F.) for samples with predominantly elongated or rounded cell in the tumor body. Patients from the “Spanish” cohort (n = 40 patients; n = 2,860 cells). Number of patients associated with each category is shown at the bottom. Representative images are shown on the side. Scale bar, 20 μm. (G) Average roundness index of melanoma cell from samples classified by the level of overall CITED1 staining. Patients from the “Spanish” cohort (n = 40; n = 2,860). Representative images of the level of CITED1 staining are shown on the side. Scale bar, 20 μm. (H) Average roundness index of melanoma cells from samples classified by level of overall CITED1 staining. Patients from the “English” cohort of patients (n = 7; n = 700). Representative images of the level of CITED1 staining are shown at the bottom. Scale bar, 20 μm. (I) (black axis) Average roundness index of melanoma cells (Pink axis) percentage of cells with CITED1 in the nucleus in the tumor body or the invasive front of primary melanomas. Patients from the “English” cohort of patients (n = 7 patients; n = 700 cells). (J) Average roundness index of melanoma cells from samples classified by percentage of nuclear CITED1. Patients from the “English” cohort of patients (n = 7; n = 700). Representative images of CITED1 nuclear staining are shown at the bottom. Scale bar, 20 μm. n.s., not significant, ^∗^p < 0.05, ^∗∗^p < 0.01, ^∗∗∗^p < 0.001. Graphs show mean ± SEM. Tukey’s post-test following one-way ANOVA (B, D, G, I, and J; left E and F), Spearman’s correlation (C), unpaired t test (H), chi-square test (right E and F). See also Figure S2.

CITED1 is an adaptor of the SMAD complex that enhances the transcription of specific SMAD2/3 target genes [9, 10] downstream of TGF-β. We found CITED1 to be downregulated after actomyosin inhibition (Figure 1E). Moreover, gene set enrichment analysis (GSEA) showed a significant enrichment in CITED1-regulated genes [33] in rounded and highly contractile cells (Figure 3A). Accordingly, we found that A375M2 rounded cells treated with ROCK inhibitors (H1152, Y27632) or compared with A375P cells not only lost roundness, but also had lower levels of CITED1 (Figure 3B). Since the functional role of CITED1 in melanoma is unclear, we decided to further investigate its connection to TGF-β-SMAD2-driven transcription.

CITED1 localization to the nucleus has been associated with its function [12, 34]. We tested the association between cell morphology and nuclear CITED1 using the panel of 16 human melanoma cell lines (Figures 3C and S2A–S2C). Cell morphology and percentage of nuclear CITED1 using immunofluorescence were assessed in cells plated on a thick layer of collagen I. The percentage of nuclear CITED1 significantly correlated with roundness (Figures 3C and S2A–S2C). We confirmed via nuclear/cytoplasmic fractionation that rounded cells (A375M2) have higher levels of nuclear CITED1 than more elongated cells (A375P) (Figure S2D). Moreover, total CITED1 levels correlated with roundness using both immunoblotting (IB) and immunofluorescence (IF) techniques (Figures S2E–S2G) (Spearman’s r = 0.86 using IB and r = 0.9 using IF).

A375M2 cells displayed the highest roundness index [25] and highest levels of nuclear CITED1 in our cell line panel (Figure 3C). Therefore, we used A375M2 cells for mouse xenograft studies. A375M2 cells were injected subcutaneously in nude mice, and after 4 weeks xenografts were excised and stained for CITED1. Each sample was scored blindly for staining intensity as negative (0), low (1), intermediate (2), or high (3). We found a correlation between rounded cell morphology and higher CITED1 levels in vivo (Figure 3D), both of which correlated with distance from the core of the tumor (Figure 3E).

We next analyzed CITED1 protein levels in two cohorts of patient samples using immunohistochemistry (Tables S1 and S2). As previously described [25, 26], we defined the tumor “invasive front” as melanoma cells with at least 50% cell surface in contact with the matrix [25]. We first took into consideration a cohort of 40 human melanoma patients (“Spanish” cohort) and analyzed melanoma cell roundness and CITED1 staining intensity. Melanoma patients with elongated cells in the tumor body were less frequent in this cohort (n = 10 patients with elongated cells in the tumor body versus n = 30 patients with rounded cells in the tumor body) (Figure 3F). However, in the former we observed significant rounding in the invasive fronts, which correlated with an increase in CITED1 staining (Figure 3F). On the other hand, melanomas with rounded cells in the tumor body displayed significantly higher levels of CITED1 when compared with tumors with elongated cells in the tumor body (Figure 3F). Across the whole cohort, we found that CITED1 levels correlated with cell roundness (Figure 3G). We then analyzed a smaller cohort of melanoma patients (“English” cohort) for a more detailed analysis of CITED1 localization. In these patients, we also observed a correlation between CITED1 staining and cell roundness (Figure 3H). Furthermore, the average roundness index of the melanoma cells increased in the invasive fronts, which correlated with percentage of nuclear CITED1 (Figures 3I and 3J).

These data illustrate how CITED1 is associated with amoeboid features in melanoma cell lines, in human xenografts, and in melanoma patients.

### SMAD2 and CITED1 Work in a Complex that Controls Amoeboid Behavior

So far, our results indicate a correlation between the expression of CITED1 and cell rounding. We therefore investigated a possible functional regulation of cell morphology and actomyosin contractility by CITED1 and the SMAD complex.

**Figure 4:**
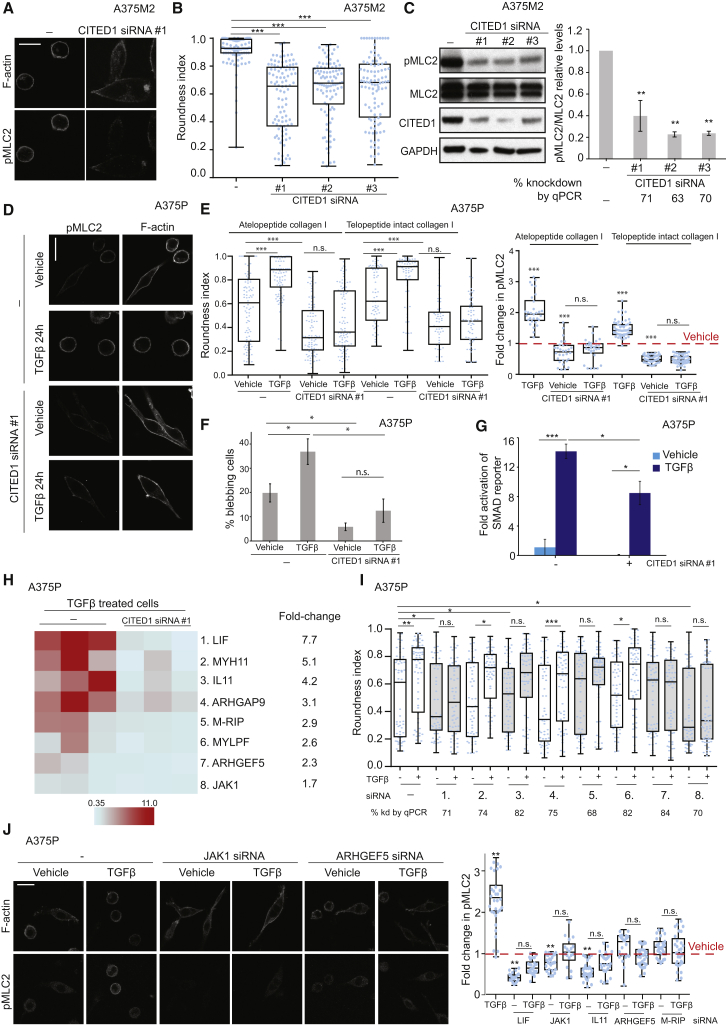
SMAD2 and CITED1 Work in a Complex that Controls Amoeboid Behavior (A) Representative confocal images of phospho-MLC2 (pMLC2) immunostaining of A375M2 cells upon CITED1siRNA SmartPool (#1) knockdown. F-actin is also shown. Scale bar, 20 μm. (B) Cell morphology (roundness index) of A375M2 cells upon CITED1 siRNA knockdown with CITED1siRNA SmartPool (#1) and 2 individual OnTargetplus oligos (#2, #3). Dots represent individual cells from three independent experiments (n = 3; n = 100). (C) Representative immunoblots (left) and quantification (right) of pMLC2, MLC2, and CITED1 of A375M2 cells on collagen I upon CITED1 siRNA knockdown (n = 3). Percentage of knockdown by qPCR is shown below. (D) Representative confocal images of pMLC2 immunostaining of A375P cells on collagen I upon CITED1 siRNA #1 knockdown and TGF-β stimulation in serum-free media. F-actin is also shown. Scale bar, 20 μm. (E) (left) Cell morphology (roundness index) of A375P cells upon CITED1 siRNA knockdown and TGF-β stimulation. (right) Quantification of pMLC2 immunostaining. Dots represent averages from three independent experiments on atelopeptide collagen I (n = 3; n = 100) and telopeptide intact collagen I (n = 3; n = 60). (F) Percentage of blebbing cells in A375P cells upon CITED1 siRNA #1 knockdown and TGF-β1 stimulation on atelopeptide collagen I (n = 3). (G) Fold activation of SMAD reporter CAGA12-CFP in A375P cells upon CITED1 siRNA #1 knockdown and after TGF-β stimulation (n = 3). (H) Heatmap representing fold decrease in gene expression after CITED1 siRNA #1 knockdown (n = 3) after TGF-β stimulation. Individual repeats are shown in the heatmap. Only genes regulated by both TGF-β and CITED1 are represented. Raw data are shown in Figure S3I. Fold decrease after CITED1 depletion is shown on the right. (I) Roundness index of A375P cells upon TGF-β treatment and knockdown of LIF, MYH11, IL11, ARHGAP9, MRIP, MYLPF, ARHGEF5, and JAK1 (n = 3; n = 100). For each gene, percentage of knockdown by qPCR is shown below graph. (J) (left) Representative confocal images of phospho-MLC2 (pMLC2) immunostaining of A375P cells after TGF-β treatment, JAK1 siRNA, and ARHGEF5 siRNA. F-actin staining is also shown. Scale bar, 20 μm. (right) Quantification of pMLC2 immunostaining of A375P cells upon TGF-β stimulation and LIF, JAK1, IL11, ARHGEF5, and MRIP knockdown. Indicated statistics are versus vehicle unless otherwise indicated by horizontal lines. n.s., not significant, ^∗^p < 0.05, ^∗∗^p < 0.01, ^∗∗∗^p < 0.001. Graphs show mean ± SEM. Tukey’s post-test following one-way ANOVA (B, C, E–H, U, and J). See also Figure S3.

Similarly to what we observed after SMAD2 knockdown, CITED1 depletion resulted in reduced cell rounding (Figures 4A and 4B), decreased MLC2 activity (Figures 4A and 4C), and loss of blebbing in A375M2 cells (Figure S3A). We verified these results using WM1361 and WM793B melanoma cell lines (Figures S3B–S3D) and using several oligonucleotides against CITED1 (Figures 4A–4C). CITED1 is therefore required to sustain actomyosin cytoskeletal activity in melanoma cells.

We next established a direct functional link between SMAD2 and CITED1. CITED1-GFP overexpression resulted in increased cell rounding (Figures S3E and S3F) and pMLC2 levels (Figures S3E and S3G). However, these effects were no longer observed if SMAD2 had been depleted from the cells via RNAi (Figures S3E–S3G). In order to establish the physical cooperation between these two factors, we performed co-immunoprecipitation studies. We were able to detect endogenous SMAD2 in immuno-precipitates isolated with HA antibodies from cells expressing HA-epitope tagged CITED1 (Figure S3H). These results show that SMAD2 and CITED1 work together in a complex to sustain actomyosin contractility and amoeboid features.

Since SMAD/CITED1 complexes act downstream of TGF-β signaling, we treated A375P melanoma cells with TGF-β in an attempt to rescue the induced amoeboid phenotype using RNAi against CITED1 (Figures 4D–4F). Interestingly, all the rounded-amoeboid features induced by TGF-β stimulation (Figure 1), such as rounding, high contractility and blebbing were ablated if CITED1 was depleted (Figures 4D–4F). These results were similar when using different types of collagen I matrix (Figure 4E). Of note, the A375P basal levels of rounding/blebbing and contractility were also reduced after CITED1 depletion, indicating that even low levels of TGF-β signaling still rely on CITED1 to control actomyosin (Figures 4D–4F).

We then investigated whether CITED1 was required for TGF-β/SMAD-driven transcriptional activation in melanoma. We did so by using a TGF-β/SMAD-dependent reporter construct (CAGA12-CFP), which measures SMAD-driven transcriptional activity via CFP expression [2]. We could confirm that A375P cells respond to TGF-β stimulation, and importantly this transcriptional response was significantly reduced after CITED1 depletion (Figure 4G). These data indicate that CITED1 is an important link between TGF-β and SMAD-driven transcription in melanoma.

We next hypothesized that TGF-β could control actomyosin force in melanoma through SMAD2/CITED1-mediated transcription. This would result in changes in the expression of genes that can alter actomyosin contractility. After a database/literature search, we selected 24 candidate genes that have been described as canonical TGF-β targets in different systems and that could potentially control cytoskeletal actomyosin dynamics (see Table S3). We further verified that these genes were regulated in our microarray (Figure 1D). After stimulation of A375P cells with TGF-β, we measured a significant increase in the mRNA levels of 17 of the genes tested (Figure S3I). Importantly, regulation of the expression of half of these genes (n = 8) was dependent on CITED1 levels (Figures 4H and S3I). These genes could be potential regulators of contractility downstream of TGF-β/CITED1 transcriptional activation.

We then individually knocked down these eight genes in A375P cells and tested the ability of the cells to respond to TGF-β treatment (Figure 4I). Importantly, depletion of five genes (JAK1, LIF, IL11, M-RIP, and ARHGEF5) reduced the ability of A375P cells to respond to TGF-β stimulation by impairing cell rounding and increased contractility levels (Figures 4I and 4J). Furthermore, depletion of JAK1 and LIF led to a significant decrease in the basal levels of rounding (Figure 4I), in accordance with basal TGF-β activity controlling basal contractile force. These results indicate that CITED1 acts downstream of TGF-β, mediating transcriptional control of several genes that promote rounding (Figure 4I) and actomyosin contractility (Figure 4J). In particular, this transcriptional regulation involves the JAK/STAT pathway (JAK1, LIF, and IL11), which is a strong regulator of contractility in melanoma [26]. As expected, loss of CITED1 resulted in reduced activation of STAT3 signaling, without affecting SMAD2 phosphorylation levels (Figure S3J). We also found that the cytoskeletal regulators ARHGEF5 and M-RIP control TGF-β-dependent contractility in our system (Figures 4I and 4J). All these data show how TGF-β promotes actomyosin contractility in melanoma through increasing transcription a set of genes in a CITED1-dependent manner.

### TGF-β-SMAD2-CITED1 Control Melanoma Detachment, Migration, and Invasion

**Figure 5:**
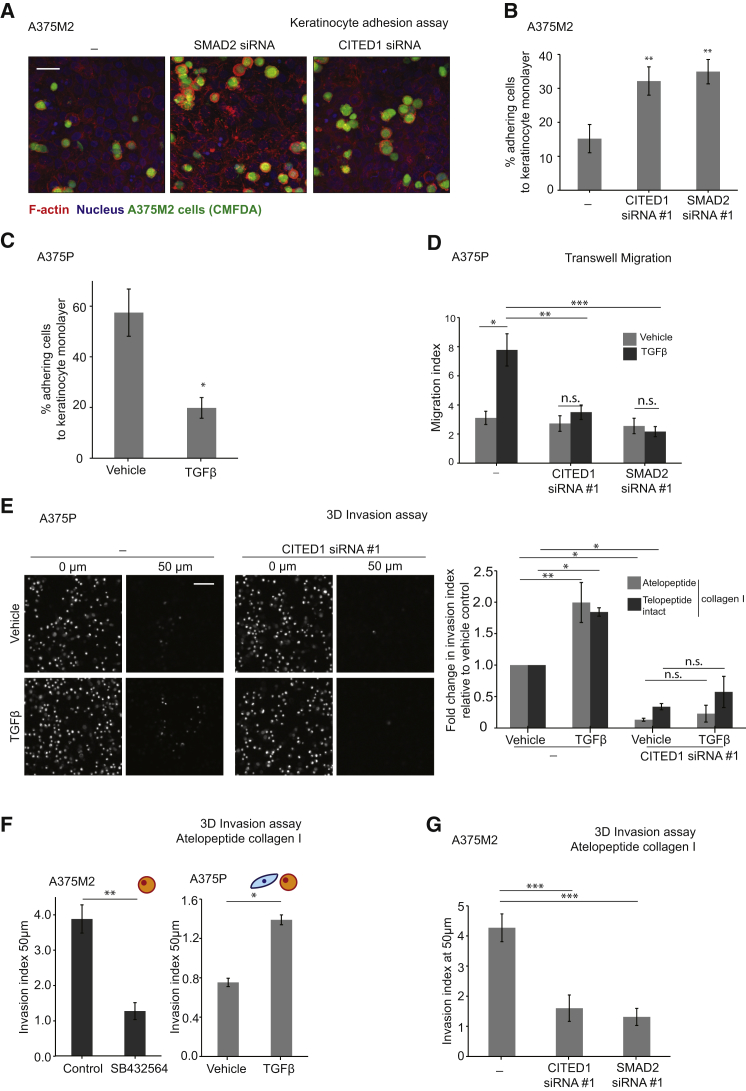
TGF-β-SMAD2-CITED1 Control Melanoma Detachment, Migration, and Invasion (A) Representative confocal images of CITED1 #1 and SMAD2 #1 siRNA-transfected A375M2 cells adhering to a monolayer of keratinocytes displayed as a maximum intensity projection. Images show F-actin staining (red), Hoechst staining (blue), and A375M2 cells (green, CMFDA staining). Scale bar, 40 μm. (B) Percentage of A375M2 cells adhering to a keratinocyte monolayer upon CITED1 or SMAD2 siRNA #1 knockdown (n = 3). (C) Percentage of A375P cells adhering to a keratinocyte monolayer. A375P cells were pretreated with TGF-β for 24 hr before the assay (n = 3). (D) Migration index for A375P cells after 4 hr of TGF-β chemotaxis and CITED1 (#1) or SMAD2 (#1) siRNA knockdown in a transwell chemotaxis assay (n = 3). (E) (left) Representative images of A375P cells invading through collagen I, showing the bottom of the well (0 μm) and a confocal slice at 50 μm within the collagen. Scale bar, 50 μm. (right) Fold change in invasion at 50 μm for A375P cells after 24 hr of TGF-β stimulation and CITED1 siRNA (#1) knockdown. Cells invading through atelopeptide collagen I (n = 4) or telopeptide intact collagen I (n = 3). (F) Invasion index at 50 μm for A375M2 upon 24 hr of SB432564 TGF-βR inhibitor treatment (left) and A375P cells after 24 hr of TGF-β stimulation (right). Cells invading through atelopeptide collagen I. (G) Invasion index at 50 μm for A375M2 cells invading though atelopeptide collagen I upon CITED1 or SMAD2 siRNA (#1) knockdown. n.s., not significant, ^∗^p < 0.05, ^∗∗^p < 0.01, ^∗∗∗^p < 0.001. Graphs show mean ± SEM. Tukey’s post-test following one-way ANOVA (B, D, E, and G), unpaired t test (C and F). See also Figure S4.

We next recapitulated the steps of melanoma progression through functional assays to determine whether TGF-β-induced transcription is functionally relevant in migration and invasion. Melanoma cells must lose interactions with keratinocytes in order to invade the dermis. Depletion of SMAD2 or CITED1 in highly contractile A375M2 cells resulted in higher levels of attachment to a monolayer of keratinocytes (Figures 5A and 5B). Conversely, addition of TGF-β to less contractile A375P cells reduced keratinocyte attachment (Figure 5C). TGF-β-treatment also reduced attachment collagen I, while CITED1 and SMAD2 depletion increased attachment to collagen I (Figures S4A–S4C). These results are in agreement with amoeboid-contractile migration being less dependent on adhesion to interstitial collagen I to allow fast migration [27].

Melanoma cells acquire the ability to migrate and invade in the vertical growth phase, we therefore performed 2D migration and 3D invasion assays. TGF-β stimulation of A375P cells resulted in increased migration (Figure 5D) and increased 3D invasion using different types of collagen I (Figures 5E, S4D, and S4E). However, depletion of CITED1 or SMAD2 impaired TGF-β-induced migration and invasion (Figures 5D and 5E), and even basal levels of invasion were decreased after CITED1 depletion (Figure 5E). Melanoma cells with intrinsically high levels of CITED1—such as A375M2—invaded efficiently in a 3D collagen matrix (Figure 5F) but displayed impaired invasion after pre-treatment with the TGF-βRI inhibitor SB431542 (Figure 5F) [35]. Conversely, addition of TGF-β to low-contractile A375P cells resulted in increased levels of invasion (Figure 5F). Furthermore, we observed a decrease in invasion of A375M2 cells if SMAD2 or CITED1 had been silenced (Figure 5G). These results suggest that TGF-β-dependent transcription controls the ability of melanoma cells to escape keratinocyte control, migrate, and invade through collagen I.

### Clinical Relevance of TGF-β-Driven Transcription in Melanoma Metastasis

**Figure 6:**
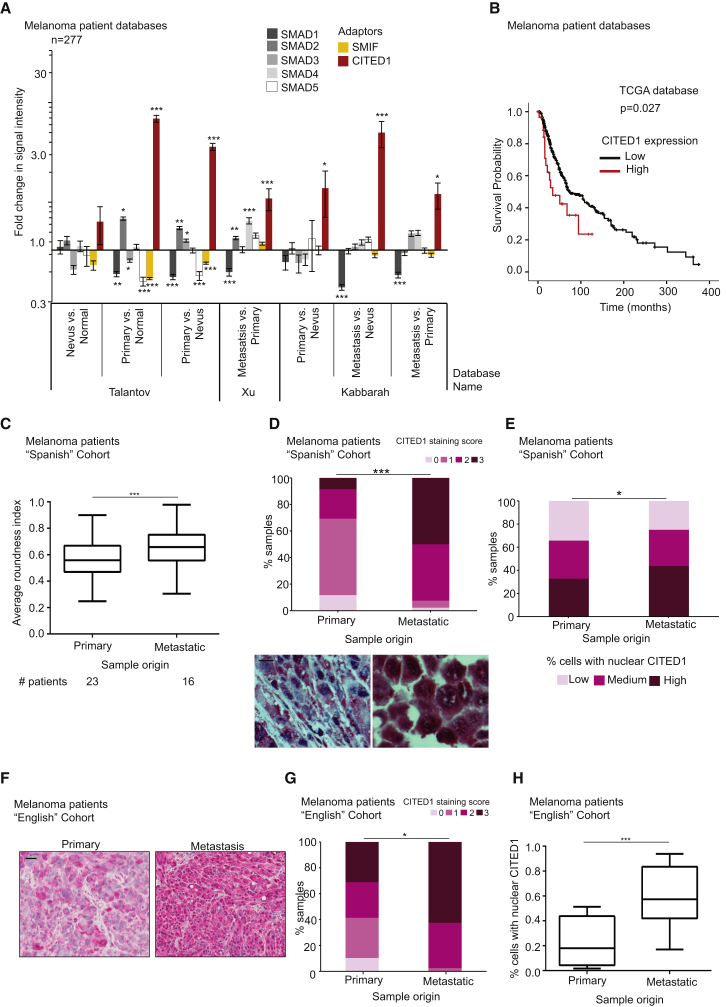
Clinical Relevance of TGF-β-Driven Transcription in Melanoma Metastasis (A) SMAD1, SMAD2, SMAD3, SMAD4, SMAD5, SMIF, and CITED1 mRNA expression (fold change) using normalized microarray gene expression data from the indicated studies (N = 277 patients). Raw data are provided in Figures S5A–S5C. (B) Kaplan-Meier estimates of overall survival in TCGA melanoma patients according to CITED1 expression. High CITED1 expression was significantly associated with poorer survival in melanoma patients (N = 354; Hazards ratio (HR), 1.81; 95% confidence interval (CI), 1.06–3.09; p = 0.027). (C) Average roundness index of melanoma cells from patients with primary or metastatic melanoma. Patients from the “Spanish” cohort of patients (n = 40 patients; n = 2,860 cells). (D) (top) Distribution of CITED1 staining in melanoma cells from patients with primary or metastatic melanoma. (bottom) Representative images of melanoma patient samples stained for CITED1 (pink) and counterstained with hematoxylin (blue). Scale bar, 20 μm. Patients from the “Spanish” cohort of patients (n = 40 patients; n = 2,860 cells). (E) Percentage of samples classified by level of nuclear localization of CITED1 in patients with primary or metastatic melanoma. Patients from the “Spanish” cohort of patients (n = 40; n = 2,860). (F) Representative images of melanoma patient samples stained for CITED1 (pink) and counterstained with hematoxylin staining (blue). Patients from the “English” cohort of patients. Scale bar, 20 μm. (G) Distribution of CITED1 staining intensity in melanoma cells in samples from patients with primary or metastatic melanoma. Patients from the “English” cohort of patients (n = 7 patients; primary n = 3, metastasis n = 4; n = 700 cells). (H) Percentage of melanoma cells with nuclear CITED1 in melanoma patient samples from (F) and (G). Patients from the “English” cohort of patients (n = 7; n = 700). n.s., not significant, ^∗^p < 0.05, ^∗∗^p < 0.01, ^∗∗∗^p < 0.001. Graphs show mean ± SEM. Tukey’s post-test following one-way ANOVA (A), Kaplan-Meier (B), unpaired t test (C and H), chi-square test (D, E, and G). See also Figure S5.

In order to understand the clinical significance of the TGF-β transcriptional network in malignant melanoma, we analyzed expression levels of the SMADs (SMAD1–5) and of the adaptor CITED1. We also analyzed levels of SMIF, another adaptor for SMAD-driven transcription [36]. We used publicly available gene expression data extracted from purified human melanoma samples derived from the Gene Expression Omnibus (GEO) to analyze mRNA expression from three independent studies: Talantov, GEO: GSE3189; Xu, GEO: GDS3966; Kabbarah, GEO: GDS1989 (Figures 6A and S5A–S5C). We found moderately increased expression of either SMAD2, SMAD3, or SMAD4 throughout progression (Figure 6A). Interestingly, the most upregulated gene was the adaptor CITED1 (Figure 6A), while the other adaptor SMIF was downregulated in some melanoma stages (Figure 6A). This suggests that the adaptor CITED1 plays a very specific role in melanoma. Throughout disease progression, CITED1 levels are strongly increased, while SMAD2/3/4 levels are only moderately upregulated (Figures S5A–S5C). This indicates that CITED1 levels may be the rate-limiting step directing specific activities of the TGF-β-SMAD transcriptional complex in melanoma. We next investigated whether CITED1 is associated with survival in melanoma patients using The Cancer Genome Atlas (TCGA) database (http://cancergenome.nih.gov/) (n = 354 patients). Interestingly, patients with high CITED1 expression had a significantly shorter overall survival than patients with low expression (Figure 6B).

The analysis of gene expression databases suggests that CITED1 mRNA is highly upregulated in metastasis. We then confirmed these findings by using IHC in human melanoma samples. In our first cohort of patients (“Spanish” cohort), we found that the average roundness of melanoma cells was increased in metastatic samples versus primary tumors (Figure 6C). This observation correlated with an increase in the total levels of CITED1 in the metastatic lesions compared to the primary tumors (Figure 6D). Furthermore, the percentage of cells with high levels of nuclear CITED1 was increased in metastatic versus primary tumors (Figure 6E). Similar results were observed in our second cohort of patients (Figures 6F–6H). These observations indicate that CITED1 is associated with poor prognosis and that its levels are higher in metastatic lesions, correlating with amoeboid cytoskeletal features in human melanoma patients.

### Functional Relevance of TGF-β-CITED1-Driven Transcription in Melanoma Metastasis In Vivo

**Figure 7:**
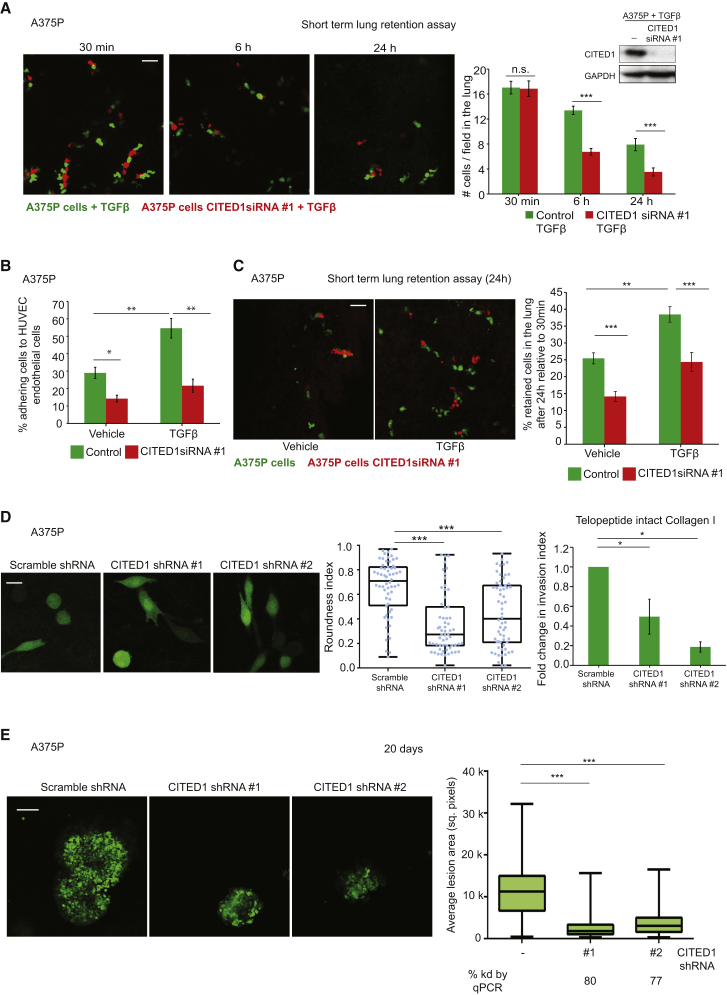
In Vivo Functional Relevance of TGF-β-Driven Transcription in Melanoma Metastasis (A) (left) Representative confocal images of mouse lungs 30 min, 6 hr, and 24 hr after tail vein co-injection of A375P cells pretreated with TGF-β (green) and pretreated with TGF-β and transfected with CITED1 siRNA (red). Scale bar, 50 μm. (right) Quantification of the number of retained cell/field in the lung after tail vein injection. (n = 20 mice). Representative immunoblot for CITED1 shown in the top-right corner. (B) Percentage of A375P cells adhering to a monolayer of HUVECs after CITED1 siRNA #1 knockdown and upon TGF-β stimulation (n = 3). (C) (left) Representative confocal images of mouse lungs 24 hr after tail vein co-injection of A375P cells (green) and transfected with CITED1 siRNA (red) with and without TGF-β pre-treatment. Scale bar, 50 μm. (right) Percentage of retained cells in the lung after tail vein injection comparing 24 hr with 30 min after injection (n = 24). (D) Representative confocal images (left) and average roundness index (right) of A375P cells stably expressing scramble of CITED1 shRNA on a thick layer of collagen I. Scale bar, 20 μm. Dots represent individual cells from three independent experiments (n = 3; n = 60). (far right) Fold change in invasion index through telopeptide intact collagen I of A375P cells stably expressing scramble or CITED1 shRNA (n = 3). Raw data are shown in Figure S6C. (E) (left) Representative confocal images of A375P cells stably expressing scramble or CITED1 shRNA forming lung colonies 20 days after tail vein injection. Scale bar, 100 μm. (right) Average lesion area for colonies formed in the lung by A375P cells 20 days of injection expressing scramble or CITED1 shRNA (n = 15). Percentage of CITED1 knockdown by qPCR is shown below. n.s., not significant, ^∗^p < 0.05, ^∗∗^p < 0.01, ^∗∗∗^p < 0.001. Graphs show mean ± SEM. Tukey’s post-test following one-way ANOVA (B–E). See also Figure S6.

As we found that CITED1 highly expressed in metastatic samples, we confirmed its functional role in supporting melanoma metastasis. One of the main sites to which melanoma metastasizes is the lung, and high actomyosin contractility in cancer cells has been reported to promote efficient lung colonization [2, 19, 25]. To test the role of CITED1 downstream of TGF-β-SMAD in metastatic colonization, we used experimental metastasis assays in mice. Tail vein injection is an accepted method to measure the ability of cancer cells to attach to blood vessels and survive in the lung [2, 19, 25]. In order to understand how early the changes in colonization ability could be observed, we compared TGF-β-treated cells with or without CITED1 in their ability to colonize the lung. Similar numbers of control or CITED1-depleted cells lodged in the lung capillaries 30 min after injection (Figure 7A). However, the number of CITED1-depleted cells that remained in the lung parenchyma after 6 and 24 hr was, respectively, 50% and 60% less than the number of control cells (Figure 7A), showing that CITED1 is important for TGF-β-mediated colonization in early stages of the process.

Furthermore, TGF-β treatment resulted in increased adhesion of A375P cells onto endothelial cells (Figure 7B), but this effect was not sustained if CITED1 had been depleted (Figure 7B). This suggests that TGF-β/CITED1-driven transcription is required for melanoma interactions with the vasculature, a crucial step in extravasation [37]. Importantly, we could confirm that TGF-β increases lung retention in melanoma 24 hr post tail vein injection, but these effects were diminished if cells had been depleted from CITED1 via small interfering RNA (siRNA) (Figure 7C). These results might be due to increased ability of melanoma cells to attach to vessels and survive in the lung parenchyma mediated by TGF-β-CITED1 activity. Interestingly, even in untreated A375P cells with low basal levels of TGF-β signaling, CITED1 deletion had a marked effect on lung colonization (Figure 7C) and melanoma adhesion to endothelia (Figure 7B). In order to understand whether this early regulation of lung colonization could eventually lead to successful tumor outgrowth over time, we generated stable cell lines with reduced levels of CITED1 using two different small hairpin RNAs (shRNAs) (Figure 7D). Morphology, contractility, and invasion levels were decreased in A375P cells depleted of CITED1 when compared to scramble shRNA (Figures 7D and S6). We analyzed the lungs 20 days after tail vein injection of these cells. Importantly, we could measure a reduction in the area of the lesions growing in the lung if cells had reduced levels of CITED1 (Figure 7E). These results indicate that as a consequence of CITED1 controlling early events in lung colonization, it is later able to control the ability of cancer cells to grow at the metastatic site.

## Discussion

TGF-β-SMAD-dependent transcription is crucial for EMT [38], metastatic dissemination, and colonization [2, 39, 40]. Melanoma is a highly metastatic cancer that arises from melanocytes [16], which derive from the neural crest and undergo EMT during development [17]. In this study, we investigated how TGF-β signaling regulates melanoma migration independently of EMT and how this connects to the actomyosin machinery. Rho-ROCK signaling in melanoma favors metastatic dissemination [19, 41, 42], but until now the direct link between the TGF-β-driven transcription and the Rho-ROCK pathway in cancer was not understood.

We have found that TGF-β is a potent regulator of the contractile actomyosin cytoskeleton in melanoma. We show that this regulation relies on SMAD/CITED1-driven transcription that sustains actomyosin contractility over time (Figure S7). To our knowledge, this is a novel function of TGF-β signaling, independent of its widely studied role in controlling EMT. Neural-crest-derived malignancies (such as schwanomas, neurofibromas, and medulloblastomas) [43] might be regulated by TGF-β in a similar fashion. Moreover, during development TGF-β promotes neural crest differentiation to highly contractile smooth muscle cells [44]. It is tempting to speculate that melanocytes retain this information and acquire the ability to become highly contractile in response to TGF-β.

On the other hand, the SMAD adaptor CITED1 was originally described to play a role in melanocyte pigmentation [9] and has been described to be upregulated in some cancers [9, 11, 12, 13, 14, 15]. CITED1 was shown to be epigenetically regulated by B-RAF^V600E^ in tumors such as melanoma and thyroid cancer [15, 45]. In the current study, we used melanoma cell lines harboring mutations in B-RAF or N-RAS (Table S4), and we only found a correlation between CITED1 nuclear levels and actomyosin contractility, independently of genetic background.

CITED1 seems to confer a metastatic advantage by promoting escape from the primary niche, migration, invasion, attachment to vessels, successful colonization, and tumor outgrowth. In particular, CITED1 regulates amoeboid migration and actomyosin contractility via a transcriptional program involving several genes, including JAK1, LIF, IL11, M-RIP, and ARHGEF5. We do not discard the possibility that other TGF-β target genes may be involved in the process, but we believe we have identified a transcriptional program regulated by CITED1 that is sufficient for sustaining actomyosin contractility over time. Furthermore, CITED1 mediates TGF-β-driven activation of STAT3, thus generating a positive feedback loop between TGF-β and JAK signaling. The role CITED1 plays in driving pro-metastatic behavior makes it an ideal candidate as a target for pharmacological intervention in melanoma. Targeted disruption of CITED1 activity or ablation of its binding to p300 or SMAD4 could be a good therapeutic opportunity.

The mesenchymal to amoeboid transition has been proposed to be independent of the activity of transcription factors, relying upon quick phenotypic adaptations to adjust to changing environmental conditions [22, 23]. Nevertheless, in the current study we propose an intriguing role for TGF-β-driven transcription in promoting amoeboid dissemination. It will be important to investigate the role of TGF-β in some developmental programs that are dependent on amoeboid migration, such as primordial germ cell migration [46], or immune responses that rely on lymphocyte amoeboid migration [47]. Since EMT can be stimulated by signals other than TGF-β (such as FGF, PDGF, Wnt, EGF, and HGF), it will also be necessary to determine whether these factors can play a role in controlling amoeboid migration.

## Experimental Procedures

### Cell Culture

WM983A, WM88, WM852, WM3060, WM3854, WM793B, and WM983B were purchased from the Wistar Collection at Coriell Cell Repository. A375P and A375M2 melanoma cells were from Dr. Richard Hynes (HHMI, MIT). SKMEL28, 501MEL, SKMEL2, WM266.4, SBCL2, WM1361, and WM1366 melanoma cells were from Professor Richard Marais (Cancer Research UK Manchester Institute). HaCat cells were from Dr. Ester Martin-Villar (Instituto de Investigaciones Biomedicas). Human umbilical vein endothelial cells (HUVECs) were from Professor Anne Ridley (King’s College London). Cells were maintained in DMEM or RPMI and kept in culture for a maximum of three to four passages.

### Cell Culture on Thick Layers of Collagen I

Collagen I matrices were prepared using telopeptide intact rat tail collagen type I (5152A, Advanced Biomatrix at 1.5 mg/ml in DMEM allowed to polymerize for 3 hr) or atelopeptide bovine collagen I (5005-B; PureCol, Advanced BioMatrix at 1.7 mg/ml in DMEM allowed to polymerize for 4 hr). Cells were imaged after 24 hr in culture in 1% serum media.

### RNAi and shRNA Sequences

All siRNA sequences were from Dharmacon. For all genes, siRNA #1 was a Smartpool (SP) and siRNAs #2 and #3 were On Target (OT) oligos from Dharmacon. When only one set of sequences is shown, Smartpool was used. For all RNAi sequences, see Supplemental Experimental Procedures.

### Quantitative Real-Time One-Step PCR

QuantiTect Primer Assays (QIAGEN) and Brilliant III SYBR Green QRT-PCR 1-step system (Agilent Technologies) were used following the manufacturer’s instructions. GAPDH was used as control.

### Immunoblotting

Cells were lysed in Laemmli sample buffer, fractionated in precast 4%–12% gradient SDS-PAGE (Life Technologies) and transferred to polyvinylidene fluoride (PVDF) filters. The ECL Plus detection Systems (GE Healthcare) with horseradish peroxidase (HRP)-conjugated secondary antibodies (GE Healthcare) were used for detection. Western blot bands were quantified using ImageJ (http://rsb.info.nih.gov/ij/). For a list of antibodies, see Supplemental Experimental Procedures.

### Animal Welfare

All animals were maintained under specific pathogen-free conditions and handled in accordance with the Institutional Committees on Animal Welfare of the UK Home Office (The Home Office Animals Scientific Procedures Act, 1986). All animal experiments were approved by the Ethical Review Process Committee at King’s College London and carried out under license from the Home Office, UK.

### Human Sample Collection and Patient Information

Patients were staged and classified according to the American Joint Committee on Cancer Melanoma Staging and Classification criteria [48]. Human samples were collected with informed written consent, in accordance with the Helsinki Declaration, and the study design was approved by the Guy’s Research Ethics Committee and Ethics Committee of Guy’s and St Thomas’ NHS Foundation Trust and the Ethics Committee of the IRBLleida Biobanc, in accordance with the Human Tissue Act, 2004. Tables S1 and S2 show clinical information from human melanoma patients.

### Immunohistochemistry

Slides were dewaxed, treated for antigen retrieval, and incubated with CITED1 antibody. Antibody detection was performed using alkaline phosphatase-conjugated secondary antibodies and developed with Dako Liquid Permanent Red Chromogen (K0640, Dako). The tumor invasive front was defined as the region of the tumor composed by only melanoma cells with at least 50% cell surface in contact with the matrix [26]. For details on quantification, see Supplemental Experimental Procedures.

### Statistical Analysis

For survival analysis, TCGA expression data were categorized using a *Z* score cutoff of 1.0. Survival curves were estimated by the Kaplan-Meier method using the log-rank test. The hazard ratio (HR) was calculated using the Cox proportional hazard model. Survival analysis was performed using SPSS (IBM). Data were plotted as a minimum-to-maximum boxplot and superimposed with dots representing single cells. Other statistical tests were performed using GraphPad Prism (GraphPad); see end of each figure legend for details. Chi-square tests were performed to compare CITED1 staining levels: when comparing elongated-core with rounded-core tumors, the data for the invasive front and the tumor core were grouped together before performing the test. Error bars indicate ±SEM.

## Author Contributions

V.S.-M. was the principal investigator who designed the study and coordinated the project, contributed to some experiments and wrote the manuscript. G.C. performed most of the experiments and contributed to the design of the study and to the writing of the manuscript. J.L.O. contributed to some experiments and the writing of the manuscript. I.R.-H. performed Kaplan-Meier survival analysis. F.O.N. and S.N.K. provided human samples for the “English” cohort and supervised lung assays performed by P.K. and J.L.O. X.M.G., O.M., and R.M.M. provided the tissue microarray of patients from the “Spanish” cohort.

## Supplemental Information

Document S1. Figures S1–S7, Tables S1–S4, and Supplemental Experimental Procedures

Document S2. Article plus Supplemental Information
