# Supplementary material for: TGF-β-Induced Transcription Sustains Amoeboid Melanoma Migration and Dissemination
Source: Curr Biol. 2015 Nov 16;25(22):2899–914. doi: 10.1016/j.cub.2015.09.054 (PMC4651903; doi:10.1016/j.cub.2015.09.054)
Supplement: Document S1. Figures S1–S7, Tables S1–S4, and Supplemental Experimental Procedures [file mmc1.pdf]

Current Biology

Supplemental Information

TGF-β-Induced Transcription Sustains Amoeboid Melanoma Migration and Dissemination

Gaia Cantelli, Jose L. Orgaz, Irene Rodriguez-Hernandez, Panagiotis Karagiannis, Oscar Maiques, Xavier Matias-Guiu, Frank O. Nestle, Rosa M. Marti, Sophia N. Karagiannis, and Victoria Sanz-Moreno

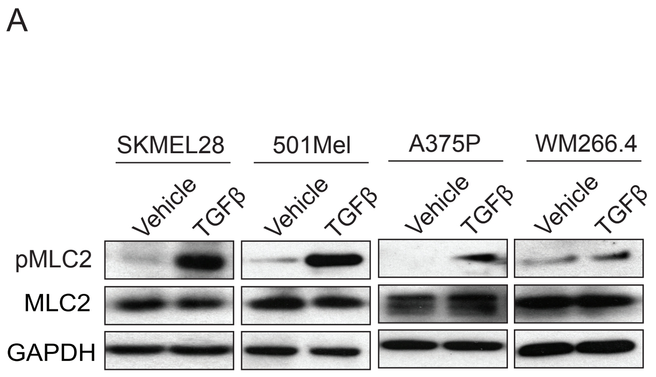
A
SKMEL28
501Mel
A375P
WM266.4
Vehicle
TGFβ
pMLC2
MLC2
GAPDH

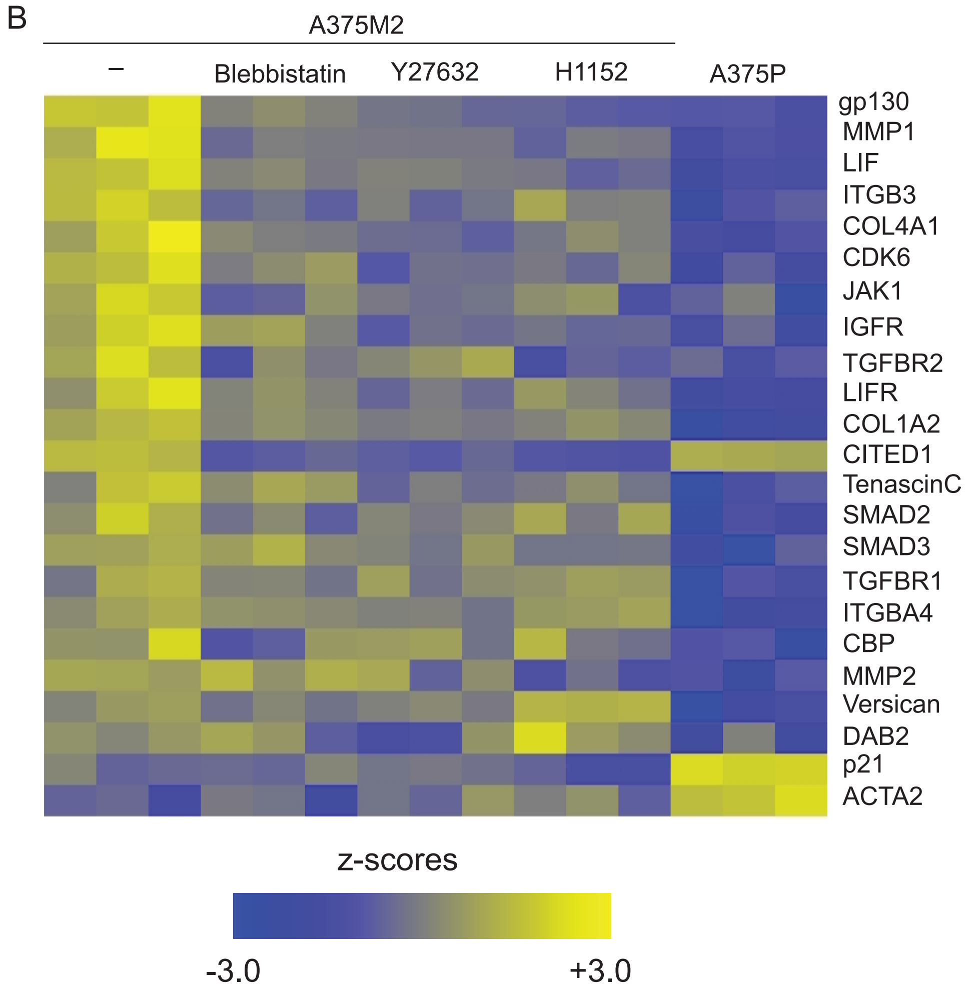
B
A375M2
–
Blebbistatin
Y27632
H1152
A375P
gp130
MMP1
LIF
ITGB3
COL4A1
CDK6
JAK1
IGFR
TGFBR2
LIFR
COL1A2
CITED1
TenascinC
SMAD2
SMAD3
TGFBR1
ITGBA4
CBP
MMP2
Versican
DAB2
p21
ACTA2
z-scores
-3.0
+3.0

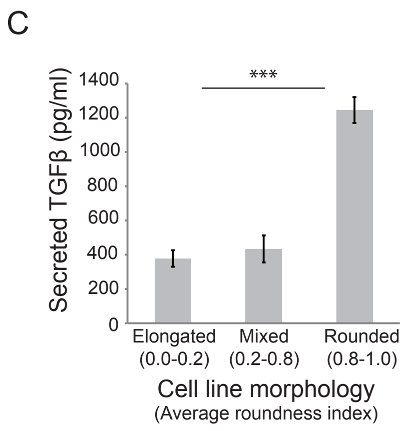
C
Secreted TGFβ (pg/ml)
1400
1200
1000
800
600
400
200
0
***
Elongated (0.0-0.2)
Mixed (0.2-0.8)
Rounded (0.8-1.0)
Cell line morphology
(Average roundness index)

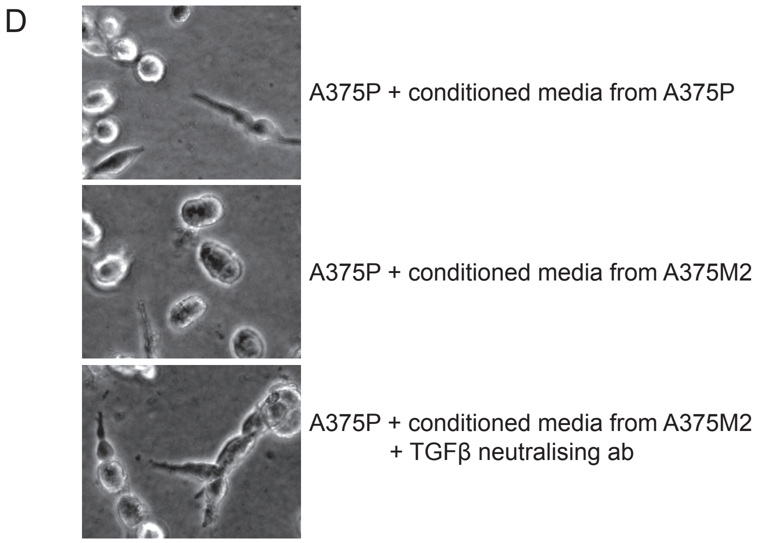
D
A375P + conditioned media from A375P
A375P + conditioned media from A375M2
A375P + conditioned media from A375M2
+ TGFβ neutralising ab

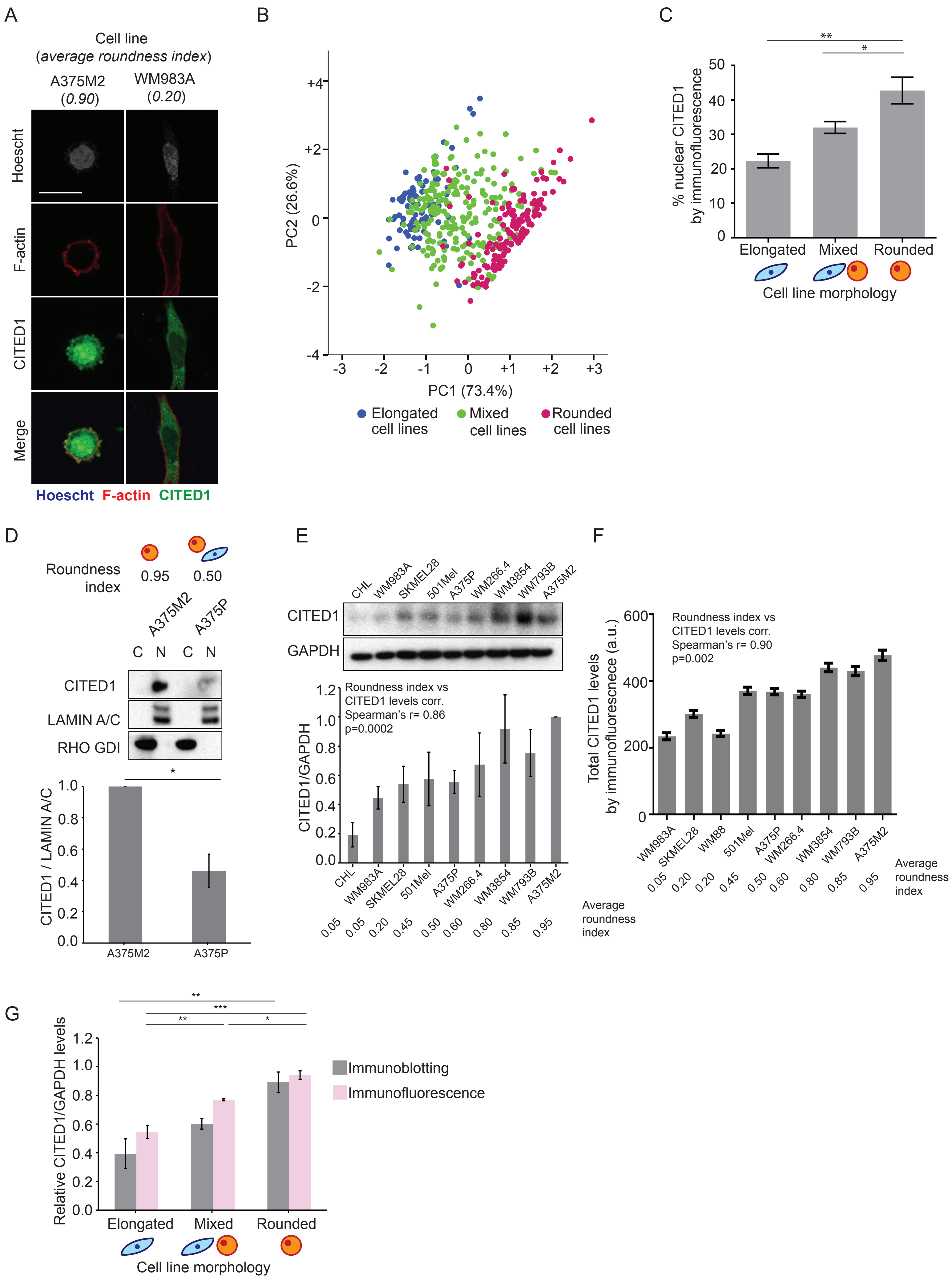
A
Cell line
(average roundness index)
A375M2 (0.90)
WM983A (0.20)
Hoescht
F-actin
CITED1
Merge
Hoescht F-actin CITED1
B
PC2 (26.6%)
PC1 (73.4%)
Elongated cell lines
Mixed cell lines
Rounded cell lines
C
% nuclear CITED1 by immunofluorescence
Elongated
Mixed
Rounded
Cell line morphology
D
Roundness index
0.95
0.50
A375M2
A375P
C N C N
CITED1
LAMIN A/C
RHO GDI
CITED1 / LAMIN A/C
E
CHL WM983A SKMEL28 501Mel A375P WM266.4 WM3854 WM793B A375M2
CITED1
GAPDH
Roundness index vs CITED1 levels corr.
Spearman's r= 0.86
p=0.0002
CITED1/GAPDH
0.05 0.05 0.20 0.45 0.50 0.60 0.80 0.85 0.95
Average roundness index
F
Roundness index vs CITED1 levels corr.
Spearman's r= 0.90
p=0.002
Total CITED1 levels by immunofluorescnece (a.u.)
WM983A SKMEL28 WM88 501Mel A375P WM266.4 WM3854 WM793B A375M2
0.05 0.20 0.20 0.45 0.50 0.60 0.80 0.85 0.95
Average roundness index
G
Relative CITED1/GAPDH levels
Immunoblotting
Immunofluorescence
Elongated
Mixed
Rounded
Cell line morphology

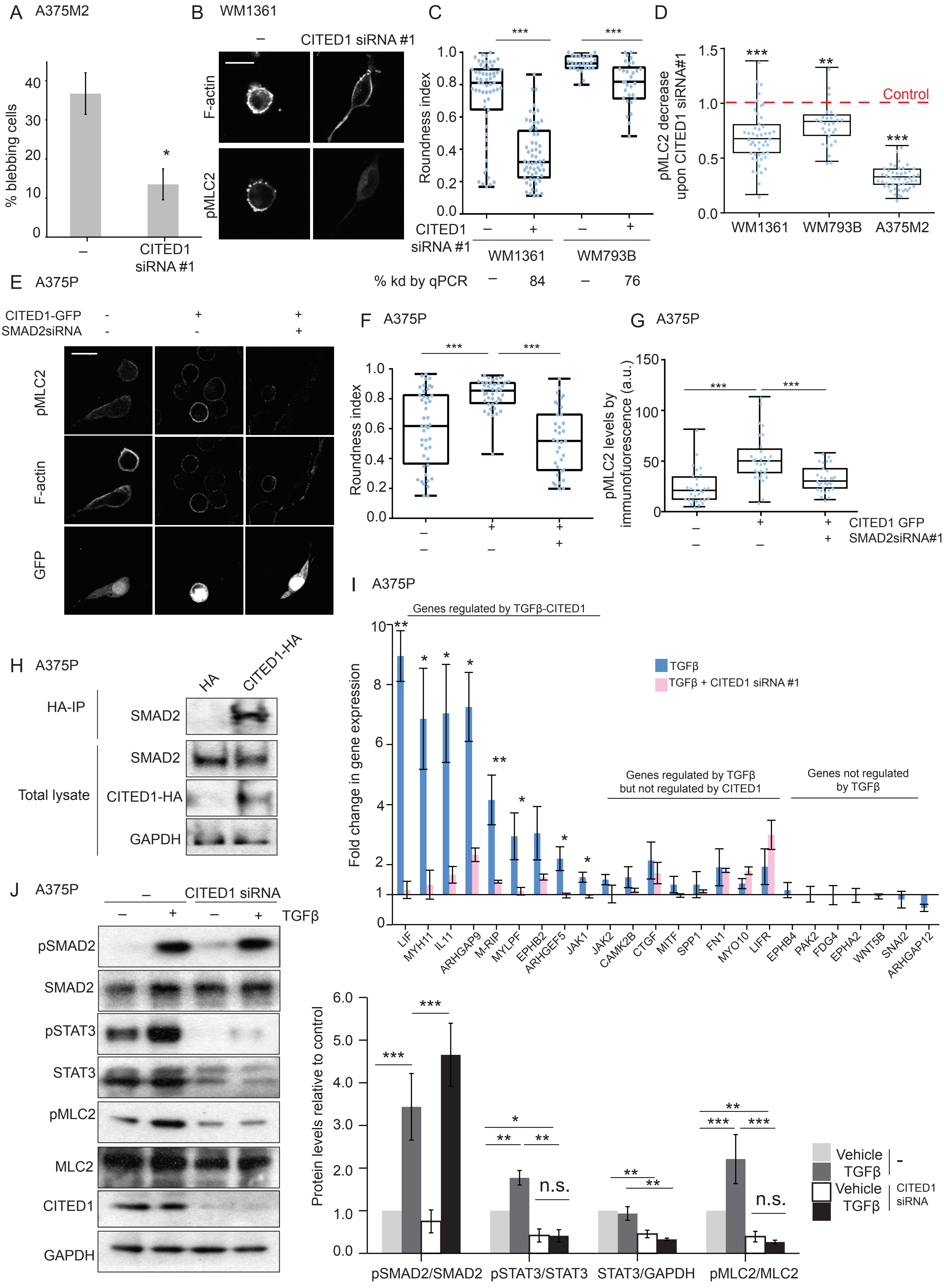

A A375M2
% blebbing cells
CITED1 siRNA #1
B WM1361
CITED1 siRNA #1
F-actin
pMLC2
C
Roundness index
CITED1 siRNA #1
WM1361
WM793B
% kd by qPCR
84
76
D
pMLC2 decrease upon CITED1 siRNA#1
Control
WM1361
WM793B
A375M2
E A375P
CITED1-GFP
SMAD2siRNA
pMLC2
F-actin
GFP
F A375P
Roundness index
G A375P
pMLC2 levels by immunofluorescence (a.u.)
CITED1 GFP
SMAD2siRNA#1
H A375P
HA
CITED1-HA
HA-IP
SMAD2
Total lysate
SMAD2
CITED1-HA
GAPDH
I A375P
Genes regulated by TGFβ-CITED1
Fold change in gene expression
TGFβ
TGFβ + CITED1 siRNA #1
Genes regulated by TGFβ but not regulated by CITED1
Genes not regulated by TGFβ
LIF
MYH11
IL11
ARHGAP9
M-RIP
MYLPF
EPHB2
ARHGEF5
JAK1
JAK2
CAMK2B
CTGF
MITF
SPP1
FN1
MYO10
LIFR
EPHB4
PAK2
FDG4
EPHA2
WNT5B
SNAI2
ARHGAP12
J A375P
CITED1 siRNA
TGFβ
pSMAD2
SMAD2
pSTAT3
STAT3
pMLC2
MLC2
CITED1
GAPDH
Protein levels relative to control
n.s.
pSMAD2/SMAD2
pSTAT3/STAT3
STAT3/GAPDH
pMLC2/MLC2
Vehicle
TGFβ
Vehicle
TGFβ
CITED1 siRNA

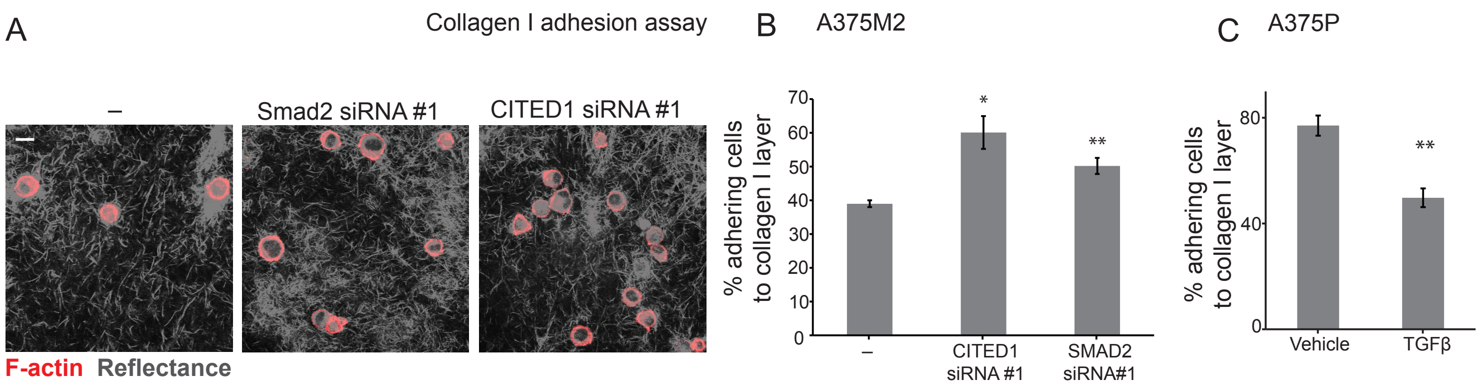
A
Collagen I adhesion assay
–
Smad2 siRNA #1
CITED1 siRNA #1
F-actin Reflectance
B
A375M2
% adhering cells
to collagen I layer
70
60
50
40
30
20
10
0
*
**
–
CITED1
siRNA #1
SMAD2
siRNA#1
C
A375P
% adhering cells
to collagen I layer
80
40
0
**
Vehicle
TGFβ

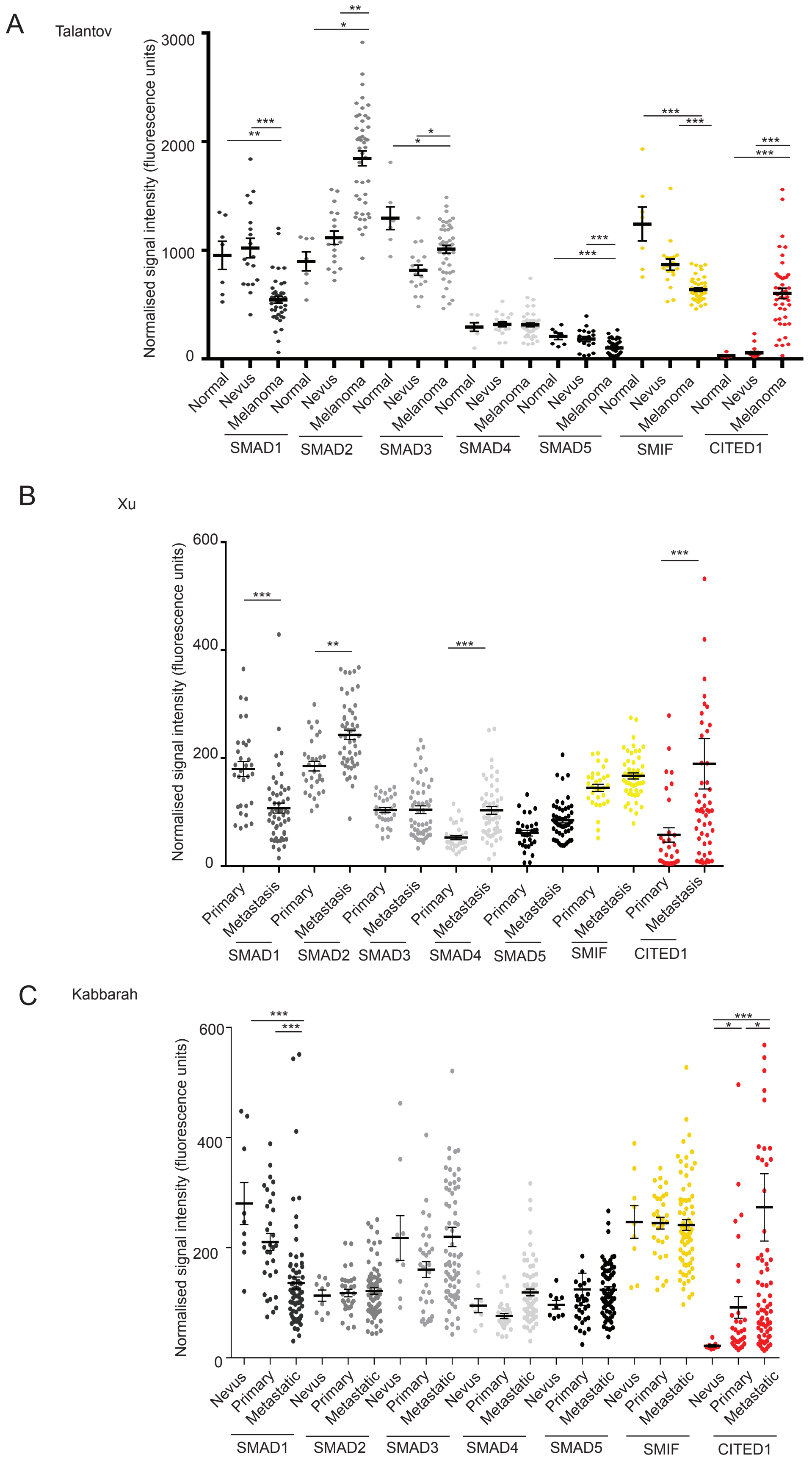
A
Talantov
Normalised signal intensity (fluorescence units)
3000
2000
1000
0
Normal
Nevus
Melanoma
SMAD1
SMAD2
SMAD3
SMAD4
SMAD5
SMIF
CITED1
B
Xu
600
400
200
Primary
Metastasis
C
Kabbarah
Primary
Metastatic

Figure S6

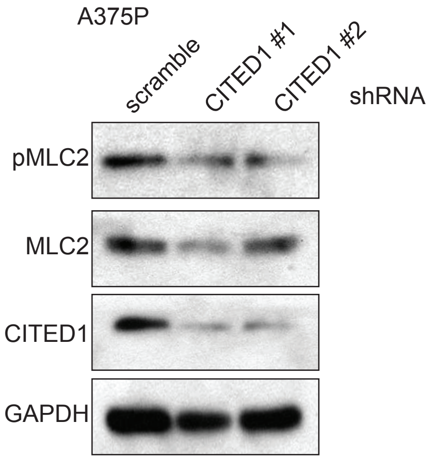
A375P
scramble
CITED1 #1
CITED1 #2
shRNA
pMLC2
MLC2
CITED1
GAPDH

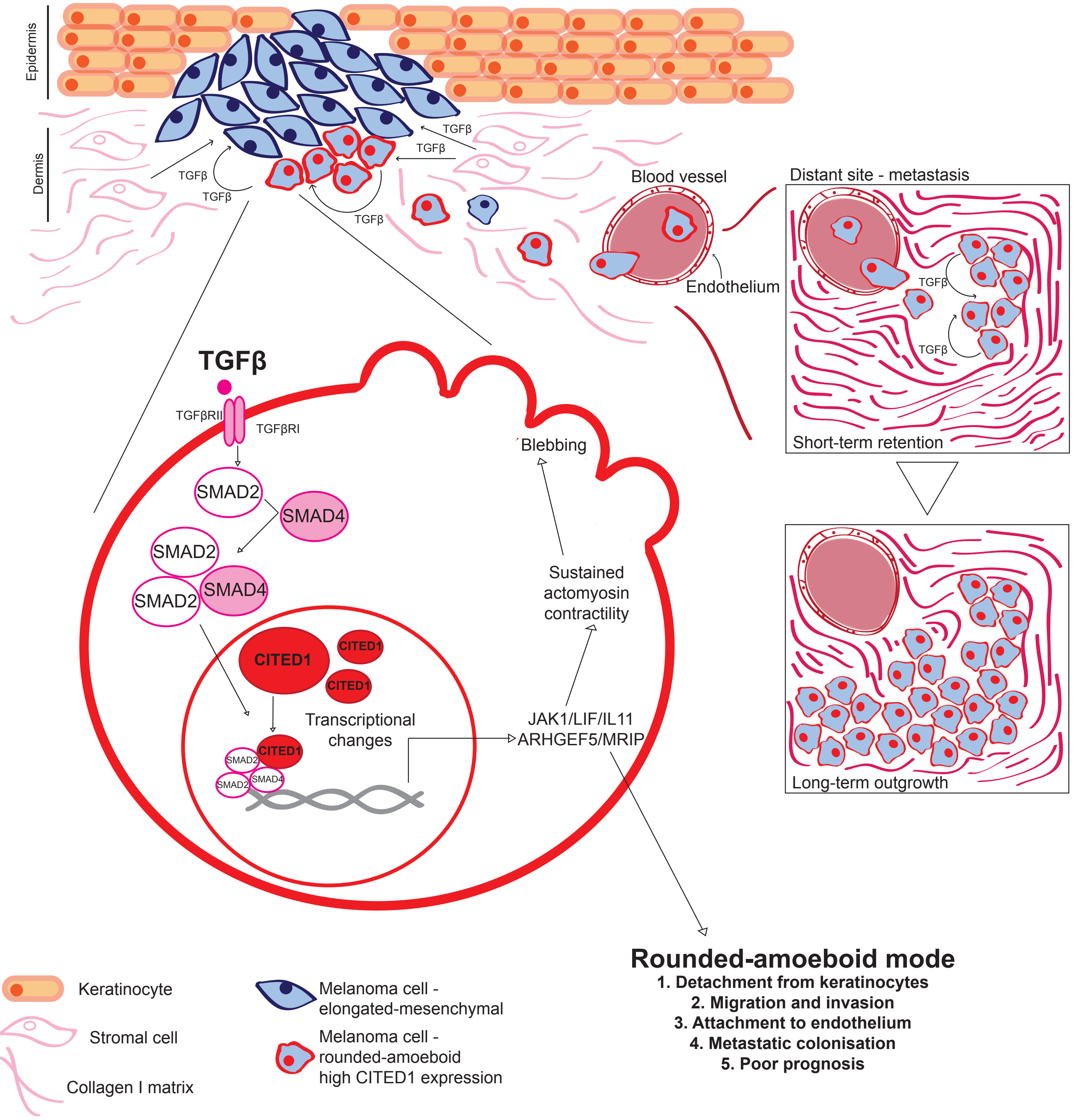
Epidermis
Dermis
TGFβ
TGFβ
TGFβ
TGFβ
TGFβ
Blood vessel
Endothelium
Distant site - metastasis
TGFβ
TGFβ
Short-term retention
Long-term outgrowth
TGFβ
TGFβRII
TGFβRI
SMAD2
SMAD4
SMAD2
SMAD2
SMAD4
CITED1
CITED1
CITED1
CITED1
SMAD2
SMAD2
SMAD4
Transcriptional
changes
Blebbing
Sustained
actomyosin
contractility
JAK1/LIF/IL11
ARHGEF5/MRIP
Rounded-amoeboid mode
1. Detachment from keratinocytes
2. Migration and invasion
3. Attachment to endothelium
4. Metastatic colonisation
5. Poor prognosis
Keratinocyte
Stromal cell
Collagen I matrix
Melanoma cell -
elongated-mesenchymal
Melanoma cell -
rounded-amoeboid
high CITED1 expression

Figure S 1 – Related to Figure 1

(A) Representative immunoblots of pMLC2 and MLC2 of SKMEL28, 501Mel, A375P and WM266.4 cells after 24 h of TGFβ stimulation.

(B) Heatmap representing the expression of all genes regulated in the network in Fig. 1E (z-scores) in A375M2 cells treated with blebbistatin, Y27632 and H1152 and in A375P cells.

(C) Levels of secreted TGFβ in the panel of cell lines grouped by cell morphology. Cells are classified as elongated (average roundness index < 0.2), mixed (average roundness index >0.2 and <0.8) and rounded (average roundness index >0.8).

(D) Representative light microscopy images of A375P cells after 24 h stimulation with conditioned media from A375P cells, A375M2 cells or A375M2 cells with TGFβ neutralising antibody. Dots represent individual cells from 3 independent experiments (n=3; N=100).

* $p<0.05$, ** $p<0.01$, *** $p<0.001$. Graphs show mean ± s.e.m. Tukey's post-test following one-way ANOVA (C).

Figure S 2 – Related to Figure 3

(A) Representative confocal images of CITED1 immunostaining of A375M2 and WM983A cells on bovine collagen I. F-actin and Hoescht staining are also shown. Scale bar, 20 μm.

(B) Principal component analysis of 16 melanoma cell lines according to the percentage of nuclear CITED1 staining and the roundness index. The horizontal axis correspond to the first principal component (PC1), accounting for 73.4% of the total variance, and vertical axis correspond to the second principal component (PC2), accounting for

26.6% of the total variance. Melanoma cell lines were coloured according to their morphology. Samples closer to each other in principal component space are similar in their roundness index and nuclear CITED1 expression.

(C) Percentage of nuclear CITED1 in the panel of cell lines grouped by cell morphology. Cells are classified as elongated (average roundness index < 0.2), mixed (average roundness index >0.2 and <0.8) and rounded (average roundness index >0.8).

(D) (Top) Representative immunoblot and (Bottom) quantification of CITED1 levels in cytoplasmic and nuclear fractions in A375M2 and A375P cells. Lamin A/C and RhoGDI are respectively markers for the nuclear and cytosolic fraction.

(E) (Top) Representative immunoblot and (Bottom) quantification of CITED1 levels in a panel of melanoma cell lines arranged according to morphology. (n=3) Correlation between average roundness index and CITED1 levels – Spearman's r=0.86

(F) Total levels of CITED1 measured by immunofluorescence in a panel of melanoma cell lines (n=3, N=100). Correlation between average roundness index and CITED1 levels – Spearman's r=0.90

(G) Relative levels of total CITED1 in cell lines grouped by cell morphology and measured by both immunoblotting and immunofluorescence (from S2E,F). Values relative to A375M2 cell line.

* p<0.05, **p<0.01, ***p<0.001. Graphs show mean ± s.e.m. Unpaired t-test (D), Spearman's correlation (E,F), Tukey's post-test following one-way ANOVA (C,G).

Figure S 3 – Related to Figure 4

(A) Percentage of blebbing cells in A375M2 cells on collagen I after CITED1 knockdown (n=3 experiments).

(B) Representative confocal images of phospho-MLC2 (pMLC2) immunostaining of WM1361 cells after CITED1knockdown in 1% serum media. F-actin is also shown. Scale bar, 20 μm.

(C) Cell morphology (roundness index) of WM1361 and WM793B cells after CITED1 knockdown. Dots represent single cells from 3 independent experiments (n=3; N=30). Percentage knockdown is shown at the bottom.

(D) Quantification of pMLC2 levels from immunostaining in confocal images from WM1361, WM793B and A375M2 cells after CITED1 knockdown. Dots represent single cells from 3 independent experiments (n=3; N=30).

(E) Representative confocal images of A375P cells CITED1-GFP overexpression (empty vector GFP was used as control) and SMAD2 knockdown in 10% serum media. Images show F-actin staining, GFP positive cells and pMLC2 staining. Scale bar 20μm. Percentage knockdown is shown at the bottom.

(F) Cell morphology (roundness index) of A375P cells on collagen I after CITED1-GFP overexpression and SMAD2 knockdown. Dots represent individual cells from 3 independent experiments (n=3; N=30).

(G) Quantification of pMLC2 levels by immunofluorescence from (E). Dots represent individual cells from 3 independent experiments (n=3; N=30).

(H) Representative immunoblot for SMAD2 and HA for HA-immunoprecipitates and total lysate of A375P cells transfected with CITED1-HA (n=3).

(I) Fold change vs control in gene expression measured by qPCR after TGFβ stimulation and TGFβ stimulation + CITED1 knockdown (n=3).

(J) Representative immunoblots (left) and quantification (right) for pSMAD2, SMAD2, GP130, pSTAT3, STAT3, pMLC2, MLC2 and CITED1 in A375P cells treated with TGFβ and depleted of CITED1 (n=3).

* $p<0.05$, ** $p<0.01$, *** $p<0.001$. Graphs show mean ± s.e.m. Tukey's post-test following one-way ANOVA (C,D,F,G,I,L), Unpaired t-test (A)

Figure S 4 – Related to Figure 5

(A) Representative confocal images of A375M2 cells adhering to a thick layer of atelopeptide collagen I. Images show F-actin (red) and reflectance showing the collagen I fibres (grey). Scale bar, 20 μm.

(B) Percentage of A375M2 cells adhering to collagen I after CITED1 or SMAD2 knockdown (n=3).

(C) Percentage of A375P cells adhering to collagen I. Cells were pretreated with TGFβ for 24 h before the assay (n=3).

* $p<0.05$, ** $p<0.01$, *** $p<0.001$. Graphs show mean ± s.e.m. Tukey's post-test following one-way ANOVA (B,D,E), Unpaired t-test (C).

Figure S 5 – Related to Figure 6

(A,B,C) SMAD1, SMAD5, SMAD2, SMAD4, SMAD3, SMIF and CITED1 mRNA expression in Talantov (a), Xu (b) and Kabbarah (c) series of melanoma tissues. Dots represent individual samples.

* $p<0.05$, ** $p<0.01$, *** $p<0.001$. Graphs show mean ± s.e.m. Tukey's post-test following one-way ANOVA (A,C), unpaired t-test (B).

Figure S 6 – Related to Figure 7

Representative immunoblot for pMLC2, MLC2 and CITED1 of A375P cells stably expressing scramble or CITED1 shRNA (See Fig. 7D,E).

Figure S7: Related to Discussion.

Model. TGFβ -induced transcription sustains amoeboid melanoma migration and dissemination

Model summarising the role of CITED1 in driving rounded-amoeboid migration. In the primary tumour, melanoma cells receive TGFβ from other cancer cells as well as from stromal cells. TGFβ signalling leads to translocation of the SMAD complex to the nucleus, where it drives CITED1-directed transcription, which supports and sustains Rho-ROCK signalling to promote actomyosin contractility and rounded-amoeboid migration. This facilitates the detachment of CITED1-expressing melanoma cells from the epithelial niche and their dissemination and outgrowth into distant sites.

Table S1 : Clinical information for human melanoma patients – English Cohort

Related to Figure 2

Patient	Gender	Age	Stage at sampling	Classification
1	M	83	IIC	Primary
2	N/A	N/A	N/A	Primary
3	N/A	N/A	N/A	Primary
4	F	84	IIIB	In transit metastasis
5	N/A	N/A	N/A	Lymph node metastasis
6	F	92	IIIB	In transit metastasis
7	M	63	IIIC	In transit metastasis

Table S2 : Clinical information for human melanoma patients– Spanish Cohort.

Related to Figure 2

Patient	Gender	Age	Classification
1	M	86	Primary
2	M	58	Primary
3	F	86	Primary
4	M	70	Primary
5	M	83	Primary
6	M	89	Primary
7	M	70	Primary
8	M	61	Primary
9	F	65	Primary
10	F	62	Primary
11	M	64	Primary
12	F	85	Primary
13	F	59	Primary
14	M	62	Primary
15	M	82	Primary
16	M	91	Primary
17	F	80	Primary
18	M	85	Primary
19	F	75	Primary
20	F	76	Primary
21	F	32	Primary
22	M	67	Primary
23	F	32	Primary
24	M	74	Metastasis
25	M	65	N/A
26	M	65	Metastasis
27	F	87	Metastasis
28	F	42	Metastasis
29	M	59	Metastasis
30	M	52	Metastasis
31	M	69	Metastasis
32	F	84	Metastasis
33	F	52	Metastasis
34	M	72	Metastasis
35	F	72	Metastasis

36	M	58	Metastasis
37	M	70	Metastasis
38	M	82	Metastasis
39	M	65	Metastasis
40	F	71	Metastasis

Table S3 : TGFβ target genes

Related to Figure 4

#	Gene name	Regulated by TGFβ (database search)	Putative cytoskeletal regulatory role (literature search)
GAPs and GEFs			
1	ARHGAP12	Adorno et al, Cell, 2009	ARHGAP12 inactivates Rac1 (Gentile et al, Oncogene, 2008). Rac1 inhibits contractility (Sanz-Moreno et al, Cell, 2008). [S1,S2]
2	ARHGAP9	Howlin et al, Oncogene, 2006	ARHGAP9 preferentially inactivates Rac1 (Furukawa et al, Biochem Biophys Res Commun, 2001). [S3]
3	ARHGEF5	Jazag et al, Oncogene, 2005	ARHGEF5 preferentially activates RhoA (Wang et al, J Biol Chem, 2009). [S4]
4	FGD4	Adorno et al, Cell, 2009	FGD4 preferentially activates Cdc42 (Ikeda et al, Oncogene, 2001). Cdc42 regulates contractility (Gadea et al, Curr Biol, 2008). [S5, S6]
LIF/JAK/STAT pathway			
5	JAK1	Howlin et al, Oncogene, 2006	Sanz-Moreno et al, Cancer Cell, 2011 [S1]
6	JAK2	Adorno et al, Cell, 2009	Sanz-Moreno et al, Cancer Cell, 2011[S1]
7	LIFR	Adorno et al, Cell, 2009	Sanz-Moreno et al, Cancer Cell, 2011[S1]
8	LIF	Adorno et al, Cell, 2009	Sanz-Moreno et al, Cancer Cell, 2011[S1]
9	IL11	Adorno et al, Cell, 2009	IL11 activtes the JAK/STAT pathway (Ernst, Clin Cancer Res., 2014), which activates contractility (Sanz-Moreno et al, Cancer Cell, 2011). [S1, S7]
Myosins and regulatory proteins			
9	MYH11	Padua et al, Cell, 2008	MYH11 regulates muscle contractility and is regulated in cancer (Alhopuro et al, PNAS, 2008) [S8]
10	MYO10	Adorno et al, Cell, 2009	MYO10 mediates the localisation of integrins (Zhang et al, Nat Cell Biol, 2004), which regulate contractility (Ahn et al, J Cell Sci, 2012) [S9, S10]
11	MYLPF	Jazag et al, Oncogene, 2005	Moh et al, J Cell Physiol, 2009 [S11]
12	M-RIP	Adorno et al, Cell, 2009	Surks et al, J Biol Chem, 2003 [S12]
Secreted factors			
13	EPHB2	Adorno et al, Cell, 2009<br>Verrecchia et al, J Biol Chem, 2001	Yang et al, J Biol Chem, 2006 [S13]
14	EPHB4	Verrecchia et al, J Biol Chem, 2001	Yang et al, J Biol Chem, 2006 [S13]
15	EPHA2	Verrecchia et al, J Biol Chem, 2001	Parri et al, J Biol Chem, 2007 [S14]
16	SPP1	Padua et al, Cell, 2008	SPP1 regulates the actin cytoskeleton (Zou et al, Cell Biochem Biophys, 2013). [S15]
17	FN1	Adorno et al, Cell, 2009	FN1 is associated with a high contractility phenotype in cancer (Sodek et al, Int J Cancer, 2009). [S16]
18	WNT5B	Adorno et al, Cell, 2009	WNT5B activates RhoA and Cdc42 (Takeshita et al, Int J Oncol, 2014). [S17]
19	CTGF	Adorno et al, Cell, 2009	CTGF is associated with a high contractility phenotype in cancer (Sodek et al, Int J Cancer, 2009). [S16]

**Kinases**			
**20**	**CAMK2B**	**Jazag et al, Oncogene, 2005**	**CAMK2B is involved in cytoskeletal remodelling (Martinez-Lozada et al, Glia, 2014) and regulation of contractility (Sossalla et al, Circ Res, 2010) in different systems [S18, S19]**
**21**	**PAK2**	**Verrecchia et al, J Biol Chem, 2001**	**Gadea et al, Curr Biol, 2008 [S5]**
**Transcription factors**			
**22**	**MITF**	**Adorno et al, Cell, 2009**	**MITF suppresses contractility in melanoma (Arozarena et al, Oncogene, 2011). [S20]**
**23**	**SNAI2**	**Adorno et al, Cell, 2009**	**SNAI2 regulates SPARC (Fenouille et al, PLoS One, 2012). SPARC deactivates Rac1 (Salvatierra et al, PLoS One, 2015). [S21, S22]**

Table S4: Genetic background and CITED1 localisation of cell lines used

Related to Discussion

Cell line	NRAS/BRAF status	% CITED1 nuclear localisation
A375M2	BRAF V600E	51
WM1366	NRAS Q61L	34
WM793B	BRAF V600E	32
WM3060	NRAS Q61K	47
WM3854	NRAS Q61L	47
Sbcl2	NRAS Q61K	42
WM1361	NRAS Q61L	31
WM266.4	BRAF V600E	25
A375P	BRAF V600E	32
501Mel	BRAF V600E	31
WM983B	BRAF V600E	32
WM852	NRAS Q61R	31
SKMEL2	NRAS Q61R	29
WM88	BRAF V600E	22
SKMEL28	BRAF V600E	18
WM983A	BRAF V600E	25
CHL	-	-

SUPPLEMENTAL EXPERIMENTAL PROCEDURES

Antibodies

Antibodies: pSer19-MLC2 (#3671), pThr18/Ser19-MLC2(#3674), pSmad2(#3101), Smad2(#3103), pY705-STAT3 (#9145), STAT3 (#9134) from Cell Signalling Technology; MLC2(sc-15370), HA(sc-7392), Lamin A/C(sc-6215) and RHOGDI(sc-360) from Santa Cruz Biotechnology; GAPDH(MAB374), CITED1(ab87978) from Abcam; CITED1(RB-9219-P) from Neomarkers.

Treatments

Cells were seeded on a thick layer of collagen I and treated with 10ng/ml human recombinant TGFβ1 (R&D, 240-B-002) for 24h in serum free media, 10μM SB431542 (Sigma, S4317-5MG), 5 μM H1152 (Calbiochem), 2.5μM Blebbistatin (Calbiochem) or 10μM Y27632 (Tocris Bioscience) for 24h.

Transfections

Cells were transfected with 20–40 nM SmartPool (SP) or individual OTs (On Targetplus) siRNA oligonucleotides (Dharmacon, see below) or with 1μg dna using Optimem-I and Lipofectamine 2000 (Invitrogen). pDONR221-CITED1 was purchased from DNASU (https://dnasu.org) and subsequently cloned into GFPpcDNA-DEST53. pMSCV-puro-HA-CITED1 was a gift from Dr WJ Muller, pGL3-CAGA12-CFP was a gift from Dr E Sahai. Cells were seeded on collagen after 48 h and imaged after 24 h in 1%-serum media.

Microarray data analysis

Network and enrichment analysis of the gene lists was performed using MetaCore from GeneGo Inc. (http://www.genego.com/metacore.php). All Metacore analysis was carried out with a threshold of 1.5. Gene enrichment analysis was performed using GSEA (http://www.broadinstitute.org/gsea/index.jsp). In all types of analysis, the comparison is carried out between A375M2 and the average of all low contractility conditions (A375M2 cells treated with H1152, Y27632 or blebbistatin and A375P cells).

Conditioned media experiments

Serum-free conditioned media was produced by A375P and A375M2 cells seeded at 250k/well for 48h. A375M2 conditioned media was treated for 10min at 37C with TGFβ neutralising antibody (1.25μg/ml; MAB1835 R&D Systems). A375P cells were seeded on a thick layer of Collagen I 12h before stimulation with conditioned media and imaged 24h after stimulation with Nikon Eclipse TX-100 with a 01QIClick FM12 camera.

Measurement of secreted TGFβ1 levels

Cells were seeded on a thick layer of collagen I and left in serum-free media for 48 h. Conditioned media was collected and levels of TGFβ1 measured. The TGF-β1 Multispecies ELISA Kit (#KAC1688, Life Sciences) was used as per manufacturer's instructions.

SMAD4 reporter assay

A375P cells were transfected with CAGA12-CFP and seeded in optical bottom plates (IBIDI, 80826). Cells were then stimulated with TGFβ for 24h, fixed and stained with Hoescht. Plates were then imaged with Zeiss LSM 510 Meta confocal microscope (Carl Zeiss, see "confocal

microscopy" section below for details). SMAD transcription activity was calculated as percentage of cells positive for CFP.

Immunohistochemistry quantification

Tumours were classified as having a predominantly "rounded" core when the average roundness index in the tumour core was greater of 0.5. For samples from the "Spanish" cohort of patients, each patient was represented in the Tissue MicroArray with eight cores: each core was imaged for 3 separate fields. For samples from the "English" cohort of patients as well as mouse xenografts, each sample was imaged for 10 separate fields. 10 representative cells were scored for roundness index. For samples from the "English" cohort and mouse xenografts, each field was scored for overall CITED1 staining. For samples from the "English" cohort, percentage of cells with nuclear CITED1 was calculated for each field. For mouse xenografts samples, the distance of each field from the edge of the xenograft was recorded. For samples from the "Spanish" cohort, each core was scored for overall CITED1 intensity as well as for approximate level of nuclear localisation of CITED1 (classed as low, intermediate or high).

Immunoprecipitation assays

A375P cells were transfected with CITED1-HA and lysed in RIPA buffer. HA epitopes were immunoprecipitated with HA-conjugated beads (Sigma, A2095) and both immunoprecipitates and total lysates were analysed via immunoblotting (see appropriate section).

Nucleo/cytosolic fractionation

Cells were lysed in cytosolic lysis buffer (40 mM HEPES, pH 7.5, 5 mM EGTA, 0.1%

Nonidet P-40, 5 mM MgCl2 1 mM DTT, 1 mM VO4,1mM benzinamide) and fractions were separated by spinning at 12000rpm. Nuclear fraction was lysed in nuclear lysis buffer (50 mM-glycerophosphate, pH 7.3, 0.2 mM EDTA, 420 mM NaCl, 1.5 mM MgCl2,1 mM DTT, 25% glycerol) and sonicated. Fractions were analysed by immunoblotting.

RNAi sequences

SMAD1

(#1: SP: 5'CCAAGAAUUUGCUCAGUUA3', 5'CAAAUGGGUUCACCUCAUA3', 5'AAACACUGGUGCUCUAUUG3', 5'CCAACAAUAAGAACCGUUU3')

SMAD2

(#1: SP: 5'GAACAAACCAGGUCUCUUG3', 5'GCAGAACUAUCUCCUACUA3', 5'GAAGAGGAGUGCGCUUAUA3', 5'GGUGUUCGAUAGCAUAUUA3'; #2: OT5: 5'GAAUUGAGCCACAGAGUAA3'; #3: OT6: 'GGUUUACUCUCCAAUGUUA3')

SMAD3

(#1: SP: 5'UCAAGAGCCUGGUCAAGAA3', 5'GAGUUCGCCUUCAAUAUGA3', 5'GGACGCAGGUUCUCCAAAC3', 5'GGACGAGGUCUGCGUGAAU3')

SMAD4

(#1: SP: 5'GUGUGCAGUUGGAAUGUAA3', 5'GUACAGAGUUACUACUUAG3',5'GAGUAUUGGUGUUCCAUUG3', 5'GUAAUGCUCCAUCAAGUAU3')

SMAD5

(#1: SP: 5’UCACAGAUCCUUCAAAUAA3’, 5’AGUCUUACCUCCAGUAUUA3’, 5’GAUCAGAUGGGUCAAGAUA3’,5’GAGUAAAUGUGUCACUAUU3’),

CITED1

(#1: SP: 5’CCAGAGCCCUGCUAUCAUC3’, 5’GGACCGAGCCAAUGAGCUU3’, 5’GAAUCACUCUCUCCUUCUG3’,5’ACUCCAACCUUGCGGUGAA3’; #2: OT7: 5’ACUCCAACCUUGCGGUGAA3’ ; #3: OT8: 5’GCCCUGCUAUCAUCGAUUC3’).

ARHGEF5

(#1: SP: 5’GCACGGAGACUCAAAGUGA3’,5’GCCAGAAGAUUGAGUUUGA3’, 5’CAAUGGCUCUUCUCUCGUU3’, 5’CUUCCAAGUAUGUGACGUA3’).

MYH11

(#1: SP: 5’GGACGUAGAGUUAUUGAAA3’,5’GGGCGGAGCUCAAUGACAA3’, 5’GCAAGAAAGACACAAGUAU3’, 5’GAAGAGACGCGGUCCCAUG3’).

MRIP

(#1: SP: 5’GUUACGAUGUCACAGAGUA3’,GUGCCACGGUGUCCGGAUA3’, GUGCACCGGUCUCGGAAAU3’, GAGGUCUUAUUGCGGGUAA3’).

ARHGAP9

(#1: SP: 5’GAACAAUGAUGUCCUGCAA3’,5’GAAGAGACCGCCCUUACAA3’, 5’GAAGGUCGGUUAGAUUUGG3’, GGGUGGUGUUAACGGGUAA3’).

IL11

(#1: SP: 5’GAGCGGACCUACUGUCCUA3’,5’GAGCCUGUGGCCAGAUACA3’, UGACAAGGCUGCGAGCGGA3’, GAACUGUGUUUGCCGCCUG3’).

MYLPF

(#1: SP: 5’AGAGGGCCAAGAGAAGGACA3’,5’UCCAGCGUCUUCUCCAUGUU3’, 5’CUGCUGACCACGCAGUGUGACC3’, 5’UACAAAAACAUCUGCUACGU3’).

JAK1

(#1: SP: 5’CCACAUAGCUGAUCUGAAA3’,5’GACAUGAUAUUGAGAACGA3’, 5’UUACAAGGAUGACGAAGGA3’, 5’CGGAUGAGGUUCUAUUUCA3’).

LIF

(#1: SP: 5’CCAAUGCCCUCUUUAUUCU3’,5’GUUCUGCACUGGAAACAUG3’, 5’GCAGAUCAUCGCCGUCUUG3’,5’GGUCUUGGCGGCAGGACUU3’).

Scramble shRNA

5’GGAATCTCATTCGATGCATAC3’

CITED1 shRNA #1

TCCTCATCCACTGGGTCCG

CITED1 shRNA #2

ATTTACAACAGAATTGGTG

Transwell migration assays

Cells were seeded at 100000 cell/well in Transwell Permeable Support plates with 8.0 μm Polycarbonate Membrane (Costar, #3422) and 10ng/μl TGFβ1 or vehicle as control was used as a chemoattractant. After 4 h at 37° C the bottom of the well was imaged (see above). Migration index was calculated as the ratio between cells seeded and the number of transmigrated cells.

3D invasion assays

Cells were resuspended in serum-free bovine collagen I or rat-tail collagen (see above for details) and spun down to the bottom of the well. 5% FCS-containing media was added on top of the gel. After 24h, plates were fixed and stained with 5 µg/ml Hoechst 33258 (Molecular probes–Life Technologies). The protocol was changed from previous publication [S1] removing the BSA coating to improve invasion levels. Plates were imaged on a Zeiss LSM 510 Meta confocal microscope (Carl Zeiss, Germany) with Zen software. The 3D migration index was calculated as number of invading cells at 50 µm divided by the total number of cells.

Adhesion assays

HaCat keratinocytes were seeded at 100000 cells/well, while HUVEC cells were seeded at 60000 cell/well; 24h later melanoma cells stained with CMFDA green dye (10 µM, Life Technologies) were seeded on top at 10000 cells/well. For collagen adhesion assays, cells were seeded on a thick layer of Collagen I. After 2h (1h for HUVEC adhesion) at 37° C cells were imaged with Nikon Eclipse TX-100 with a 01QIClick FM12 camera and Nikon C-SHO fluorescence lamp. Plates were washed twice with PBS and re-imaged. Percentage of adhering cells was calculated as the number of cells imaged after vs before washing.

Confocal fluorescence microscopy and image quantification

Cells were seeded on top of a collagen I matrix [33], fixed, permeabilized and blocked in 5% BSA. Then cells were immunostained with specific antibodies (see above), which were detected with secondary Alexa Fluor 488, 546 or 647 antibodies (Life Technologies). F-actin and nuclear staining were performed Alexa Fluor 546-phalloidin (Life Technologies) and 5µg/ml Hoechst 33258 (Molecular probes–Life Technologies). Imaging was carried out on a Zeiss LSM 510 Meta confocal microscope (Carl Zeiss) with C-Apochromat 40/1.2NA (water) objective lenses and Zen software (Carl Zeiss). ImageJ was used to assess cell morphology (roundness-index) and pMLC2 intensity (pixel intensity in single cells relative to the cell area).

Analysis of gene expression from human databases

From the public database GEO (Gene Expression Omnibus) we extracted the Talantov: GSE3189, Xu: GDS3966) and Kabbarah: GDS1989 series and analysed expression of SMADs, SMIF and CITED1, for a total of 277 patients. Data were normalized using Gene Pattern (http://www.broadinstitute.org/cancer/software/genepattern/).

Gene expression data of 354 human melanoma samples from The Cancer Genome Atlas (TCGA) database (http://cancergenome.nih.gov/) was used to analyse CITED1 expression in melanoma outcome. We only considered samples with >70% tumour cell content from non neo-adjuvant treated patients.

Mouse xenografts

Nude mice were injected subcutaneously with $1x10^6$ GFP-A375M2 cells and killed after 1 month. Tumours were excised, paraffin-embedded and stained (see immunohistochemistry).

Lung retention assays

A375P cells were transfected with CITED1 siRNA #1: 48h later they were treated with TGFβ and 24h later cells were co-injected into the tail vein of NOD/SCID/ IL-2Rγ-/- mice (NSG, Charles River) (male and female between 6–12 weeks old; mice were age- and sex-matched between the groups). Immediately prior to injection, cells were labelled with 10μM CMFDA-Green (C7025, Life Technologies) or 15μM CMRA-Orange (C34551, Life Technologies). The experiment was repeated twice (n=2) with inverted dye colours. Mice were sacrificed after 30min, 6h and 24h and the lungs were excised, washed, fixed and examined under a confocal microscope.

Lung outgrowth assays

A375P stable cell lines were generated expressing GFP and scramble or shRNAs against CITED1. These cells were injected in the tail vein of NOD/SCID/ IL-2Rγ-/- mice (see above). Mice were sacrificed after 20 days and the lungs were excised, washed, fixed and examined under a confocal microscope.

SUPPLEMENTARY REFERENCES

S1. Sanz-Moreno, V., Gaggioli, C., Yeo, M., Albrengues, J., Wallberg, F., Viros, A., Hooper, S., Mitter, R., Feral, C.C., Cook, M., et al. (2011). ROCK and JAK1 signaling cooperate to control actomyosin contractility in tumor cells and stroma. Cancer cell *20*, 229-245.

S2. Gentile, A., D'Alessandro, L., Lazzari, L., Martinoglio, B., Bertotti, A., Mira, A., Lanzetti, L., Comoglio, P.M., and Medico, E. (2008). Met-driven invasive growth involves transcriptional regulation of Arhgap12. Oncogene *27*, 5590-5598.

S3. Furukawa, Y., Kawasoe, T., Daigo, Y., Nishiwaki, T., Ishiguro, H., Takahashi, M., Kitayama, J., and Nakamura, Y. (2001). Isolation of a novel human gene, ARHGAP9, encoding a rho-GTPase activating protein. Biochemical and biophysical research communications *284*, 643-649.

S4. Wang, Z., Kumamoto, Y., Wang, P., Gan, X., Lehmann, D., Smrcka, A.V., Cohn, L., Iwasaki, A., Li, L., and Wu, D. (2009). Regulation of immature dendritic cell migration by RhoA guanine nucleotide exchange factor Arhgef5. The Journal of biological chemistry *284*, 28599-28606.

S5. Gadea, G., Sanz-Moreno, V., Self, A., Godi, A., and Marshall, C.J. (2008). DOCK10-mediated Cdc42 activation is necessary for amoeboid invasion of melanoma cells. Current biology : CB *18*, 1456-1465.

S6. Ikeda, W., Nakanishi, H., Tanaka, Y., Tachibana, K., and Takai, Y. (2001). Cooperation of Cdc42 small G protein-activating and actin filament-binding activities of frabin in microspike formation. Oncogene *20*, 3457-3463.

S7. Ernst, M., and Putoczki, T.L. (2014). Molecular pathways: IL11 as a tumor-promoting cytokine-translational implications for cancers. Clinical cancer research : an official journal of the American Association for Cancer Research *20*, 5579-5588.

S8. Alhopuro, P., Phichith, D., Tuupanen, S., Sammalkorpi, H., Nybondas, M., Saharinen, J., Robinson, J.P., Yang, Z., Chen, L.Q., Orntoft, T., et al. (2008). Unregulated smooth-muscle myosin in human intestinal neoplasia. Proceedings of the National Academy of Sciences of the United States of America *105*, 5513-5518.

S9. Zhang, H., Berg, J.S., Li, Z., Wang, Y., Lang, P., Sousa, A.D., Bhaskar, A., Cheney, R.E., and Stromblad, S. (2004). Myosin-X provides a motor-based link between integrins and the cytoskeleton. Nature cell biology *6*, 523-531.

S10. Ahn, J., Sanz-Moreno, V., and Marshall, C.J. (2012). The metastasis gene NEDD9 product acts through integrin beta3 and Src to promote mesenchymal motility and inhibit amoeboid motility. Journal of cell science *125*, 1814-1826.

S11. Moh, M.C., Tian, Q., Zhang, T., Lee, L.H., and Shen, S. (2009). The immunoglobulin-like cell adhesion molecule hepaCAM modulates cell adhesion and motility through direct interaction with the actin cytoskeleton. Journal of cellular physiology *219*, 382-391.

S12. Surks, H.K., Richards, C.T., and Mendelsohn, M.E. (2003). Myosin phosphatase-Rho interacting protein. A new member of the myosin phosphatase complex that directly binds RhoA. The Journal of biological chemistry *278*, 51484-51493.

S13. Yang, N.Y., Pasquale, E.B., Owen, L.B., and Ethell, I.M. (2006). The EphB4 receptor-tyrosine kinase promotes the migration of melanoma cells through Rho-mediated actin cytoskeleton reorganization. The Journal of biological chemistry *281*, 32574-32586.

S14. Parri, M., Buricchi, F., Giannoni, E., Grimaldi, G., Mello, T., Raugei, G., Ramponi, G., and Chiarugi, P. (2007). EphrinA1 activates a Src/focal adhesion kinase-mediated motility response leading to rho-dependent actino/myosin contractility. The Journal of biological chemistry *282*, 19619-19628.

S15. Zou, C., Luo, Q., Qin, J., Shi, Y., Yang, L., Ju, B., and Song, G. (2013). Osteopontin promotes mesenchymal stem cell migration and lessens cell stiffness via integrin beta1, FAK, and ERK pathways. Cell biochemistry and biophysics *65*, 455-462.

S16. Sodek, K.L., Ringuette, M.J., and Brown, T.J. (2009). Compact spheroid formation by ovarian cancer cells is associated with contractile behavior and an invasive phenotype. International journal of cancer. Journal international du cancer *124*, 2060-2070.
S17. Takeshita, A., Iwai, S., Morita, Y., Niki-Yonekawa, A., Hamada, M., and Yura, Y. (2014). Wnt5b promotes the cell motility essential for metastasis of oral squamous cell carcinoma through active Cdc42 and RhoA. International journal of oncology *44*, 59-68.
S18. Martinez-Lozada, Z., Waggener, C.T., Kim, K., Zou, S., Knapp, P.E., Hayashi, Y., Ortega, A., and Fuss, B. (2014). Activation of sodium-dependent glutamate transporters regulates the morphological aspects of oligodendrocyte maturation via signaling through calcium/calmodulin-dependent kinase IIbeta's actin-binding/-stabilizing domain. Glia *62*, 1543-1558.
S19. Sossalla, S., Fluschnik, N., Schotola, H., Ort, K.R., Neef, S., Schulte, T., Wittkopper, K., Renner, A., Schmitto, J.D., Gummert, J., et al. (2010). Inhibition of elevated Ca2+/calmodulin-dependent protein kinase II improves contractility in human failing myocardium. Circulation research *107*, 1150-1161.
S20. Arozarena, I., Bischof, H., Gilby, D., Belloni, B., Dummer, R., and Wellbrock, C. (2011). In melanoma, beta-catenin is a suppressor of invasion. Oncogene *30*, 4531-4543.
S21. Fenouille, N., Tichet, M., Dufies, M., Pottier, A., Mogha, A., Soo, J.K., Rocchi, S., Mallavialle, A., Galibert, M.D., Khammari, A., et al. (2012). The epithelial-mesenchymal transition (EMT) regulatory factor SLUG (SNAI2) is a downstream target of SPARC and AKT in promoting melanoma cell invasion. PloS one *7*, e40378.
S22. Salvatierra, E., Alvarez, M.J., Leishman, C.C., Rivas Baquero, E., Lutzky, V.P., Chuluyan, H.E., and Podhajcer, O.L. (2015). SPARC Controls Melanoma Cell Plasticity through Rac1. PloS one *10*, e0134714.
